# Origin of class B J-domain proteins involved in amyloid transactions

**DOI:** 10.1073/pnas.2522403123

**Published:** 2026-01-09

**Authors:** Przemyslaw Domanski, Milena Stolarska, Katarzyna Kalinowska, Dominik Purzycki, Brenda A. Schilke, Hubert Wyszkowski, Marcin Pitek, Aneta Szymanska, Katarzyna Bury, Jacek Czub, Agnieszka A. Klosowska, Elizabeth A. Craig, Jaroslaw Marszalek, Bartlomiej Tomiczek

**Affiliations:** ^a^Intercollegiate Faculty of Biotechnology, University of Gdansk, Gdansk 80-307, Poland; ^b^Department of Physical Chemistry, Gdansk University of Technology, Gdansk 80-233, Poland; ^c^Department of Biochemistry, University of Wisconsin-Madison, Madison, WI 53726; ^d^Faculty of Chemistry, University of Gdansk, Gdansk 80-308, Poland

**Keywords:** molecular chaperones, protein disaggregation, ancestral protein resurrection, convergent evolution, protein family evolution

## Abstract

J-domain protein (JDP) chaperones function widely in proteostasis. Notably, eukaryotic class B JDPs of the cytosol/nucleus prevent assembly or drive disassembly of amyloid aggregates known to cause neurodegenerative diseases, yet their evolutionary origin is not known. Members of the most ubiquitous class B subgroup, canonical B (B^C^) JDPs, lack the signature zinc finger domain (ZnF) of the more prevalent class A JDPs, while having other key features in common. Our phylogenetic analysis revealed that B^C^ JDPs evolved more than once from class A duplicates, losing their ZnF. The cytonuclear B^C^s emerged at the base of eukaryotes. Cytonuclear class B’ (i.e., B’^(ST)^) JDPs that have a substrate binding domain of unknown origin, distinct from that of As and B^C^s, emerged from a B^C^ duplication at the base of metazoans and subsequently multiplied by duplications. The origin of B’^(ST)^s, which are capable of suppressing formation of amyloid aggregates, predated the emergence of disease-causing amyloidogenic proteins. Using ancestral protein resurrection, we tested when cytonuclear Bs evolved their amyloid related functions. We found that their common ancestor with As, AncAB that has a ZnF does not efficiently facilitate disassembly of amyloid fibrils, while AncB, which lacks a ZnF, does. Overall, our findings are consistent with the idea that, though the ZnF of class A JDPs is important for some roles, its loss allowed evolution of novel functions, as illustrated by the ability of B^C^ and B’^(ST)^ JDPs to control amyloid aggregate levels.

## Significance

Across prokaryotes and eukaryotes, J-domain proteins (JDPs) are key players in Hsp70 chaperone systems that maintain cellular protein homeostasis. The abundant class A and B JDPs have structural similarities, yet their origin has remained unresolved. Here, we show that B JDPs independently evolved from As more than once. In each case, A lost its zinc finger domain (ZnF), suggesting that such loss allowed evolution of new functions. Supporting this idea, biochemical resurrection of an ancestral eukaryotic B revealed that its ability to disassemble amyloid aggregates, differentiating it from As, evolved after ZnF loss. Later, the subset of Bs implicated in suppression of disease-causing amyloid aggregate formation originated from a duplicate of this B in an ancestor of animals.

J-domain proteins (JDPs) are obligatory cochaperones of Hsp70s. Together, JDP/Hsp70 systems function in many cellular processes—from protein homeostasis, including folding of polypeptide chains and maintenance of protein structure upon stress, to remodeling of protein complexes and aggregates, including amyloids responsible for human disease (1–8). Interaction of Hsp70 with a substrate polypeptide is critical for all these functions (9). All JDPs have a J-domain that interacts with Hsp70, stimulating its inherently low ATPase activity, thereby stabilizing substrate interaction (10, 11). Many, including the first identified and best-studied JDP, DnaJ of *Escherichia coli*, also bind substrates (12, 13). Via the J-domain–Hsp70 interaction, these JDPs “deliver” their bound substrates to their Hsp70 partners (14). Together with a nucleotide exchange factor (NEF), JDPs and Hsp70s form systems for efficient cycles of interaction with substrate protein. By prioritizing substrate interaction in this way, JDPs can be said to “direct” Hsp70s to perform specific function(s) in cells (1, 2, 6, 7).

**Fig. 1.**
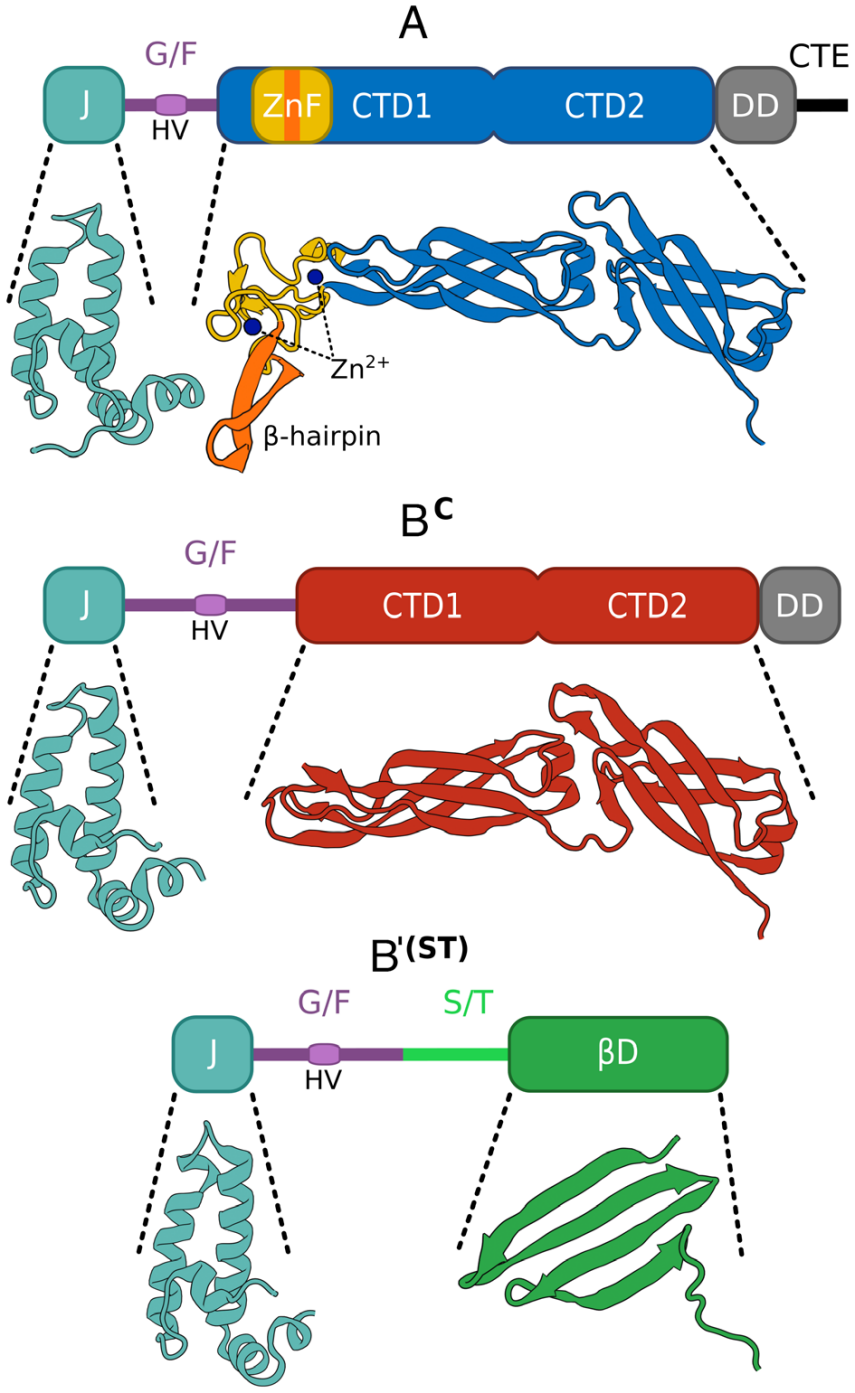
Domain organization of class A and B JDPs. Line diagrams of three classes of human JDPs: A, *Top*; B^C^, middle; B’^(ST)^, *Bottom* (structured domains, rectangles; unstructured regions, lines) with structures of J-domains and substrate binding domains shown. All three have an N-terminal J-domain (J; cyan) followed by a disordered glycine/phenylalanine rich region (G/F; violet) that contains a short helical segment termed helix V (HV). Both A and B^C^ JDPs have two C-terminal substrate binding domains (CTD1 and CTD2; blue, Class A and red, Class B) and a dimerization domain (DD; gray) after the G/F region. Class A members have a defining zinc finger domain (ZnF; yellow/orange) extending from CTD1, as well as a C-terminal extension (CTE; black) following the DD. B’^(ST)^s contain an unstructured serine/threonine rich segment (ST; light green) followed by a substrate binding β-pleated sheet domain (βD; green). Structures: DNAJA2-J (PDB: 2lo1), CTD1/2 (PDB: 7zhs); DNAJB1-J (PDB: 6z5n), CTD1/2 (PDB: 3agx); DNAJB6-J (PDB: 6u3r), βD (PDB: 7jsq).

Hundreds of structurally and functionally divergent JDPs have been identified across the tree of life (15–17). Most, however, have a limited phylogenetic distribution, being present in subsets of bacterial, archaeal, or eukaryotic lineages. Orthologs of DnaJ of *E. coli*, have by far the broadest phylogenetic distribution. Called class A or type I JDPs, they are present in most bacterial, in some archaeal, and in the vast majority of eukaryotic lineages (18, 19). In eukaryotic cells they are present in all major subcellular compartments: mitochondria, plastids, the endoplasmic reticulum (ER), and the cytosol/nucleus (C/N) (15). Class A JDPs are structurally complex (Fig. 1), having an N-terminal J-domain, an adjacent glycine/phenylalanine rich (G/F) region, followed by two β-sandwich substrate binding domains (CTD1 and CTD2), a dimerization domain (DD), and a C-terminal extension (CTE). All class A JDPs have a zinc finger domain (ZnF) protruding from the CTD1—their diagnostic feature (Fig. 1) (18). The ZnF is known to be involved in substrate interactions (13, 20, 21).

In both bacteria and compartments of eukaryotic cells, Class A JDPs are often accompanied by JDPs of the B class (type II). By definition, class B, like class A, JDPs have an N-terminal J-domain and an adjacent G/F region, but lack a ZnF (18). Class B JDPs can be divided into two types. Some, termed canonical B (B^C^), such as CbpA of *E. coli*, share the substrate binding CTD1/2 and dimerization domains (DD) with class A members (Fig. 1). Others, which we refer to throughout as B’ JDPs, do not have CTD1/2 or DD. The best-studied B’ JDPs are those that are found in the cytosol/nucleus of metazoans and have a serine/threonine rich segment (ST) followed by a substrate binding β-pleated sheet domain (βD) immediately after the G/F region (6). These are referred to as B’^(ST)^ throughout (Fig. 1). Class A and B JDPs are involved in general chaperone functions, including refolding of unfolded proteins (1, 7, 22, 23). But each, upon interacting with specific substrates, drives distinct processes. For example, class A JDPs, via their ZnF, prevent aggregation of substrates with unstable β-sheets (e.g., mutant p53 oncoprotein) (21), while Bs are more potent in disaggregation of amorphous protein aggregates (24, 25) and have been repeatedly tied to amyloids (6, 26).

Amyloids, highly ordered β-sheet-rich protein fibrils, are found in all domains of life (27–29). In some cases, amyloids play a functional role (e.g., polypeptide hormones are stored in a β-sheet conformation; bacteria use β-sheet fibrils in biofilms). In other cases, amyloids are pathogenic (e.g., prions cause Creutzfeldt–Jakob disease). In fungi, B^C^ JDPs (i.e., Sis1 in *Saccharomyces cerevisiae*) are an essential part of the system that fragments many types of yeast prions enabling their passage from one generation to the next (26). In humans, amyloids are associated with a variety of disease states, particularly neurodegenerative disorders including Alzheimer’s, Parkinson’s, and Huntington’s diseases. Human B^C^ JDPs (i.e., DNAJB1) drive Hsp70 dependent, amyloid disaggregation, while B’^(ST)^ JDPs (i.e., DNAJB6 and B8) are particularly potent in preventing amyloid fibrils formation (6, 7, 30–32).

While recent research has provided much new information about the functional similarities and differences among JDPs from diverse systems, the fundamental question of the origin of JDPs, particularly B^C^s and B’^(ST)^s, has not been addressed. Because such information is relevant to understanding the origins of functional differences between class A and B JDPs and the ability of class B JDPs to affect amyloids, we carried out extensive phylogenetic analyses. Our results revealed that B^C^s evolved independently more than once from class A ancestors, convergently losing their ZnF. Using biochemical resurrection of inferred ancestral sequences, we found that the ability of cytonuclear class B^C^ JDPs to disassemble amyloid fibrils emerged early, but subsequent to ZnF loss.

## Results and Discussion

### Class B^C^ JDPs Evolved Independently More Than Once From Class A Ancestors.

**Fig. 2.**
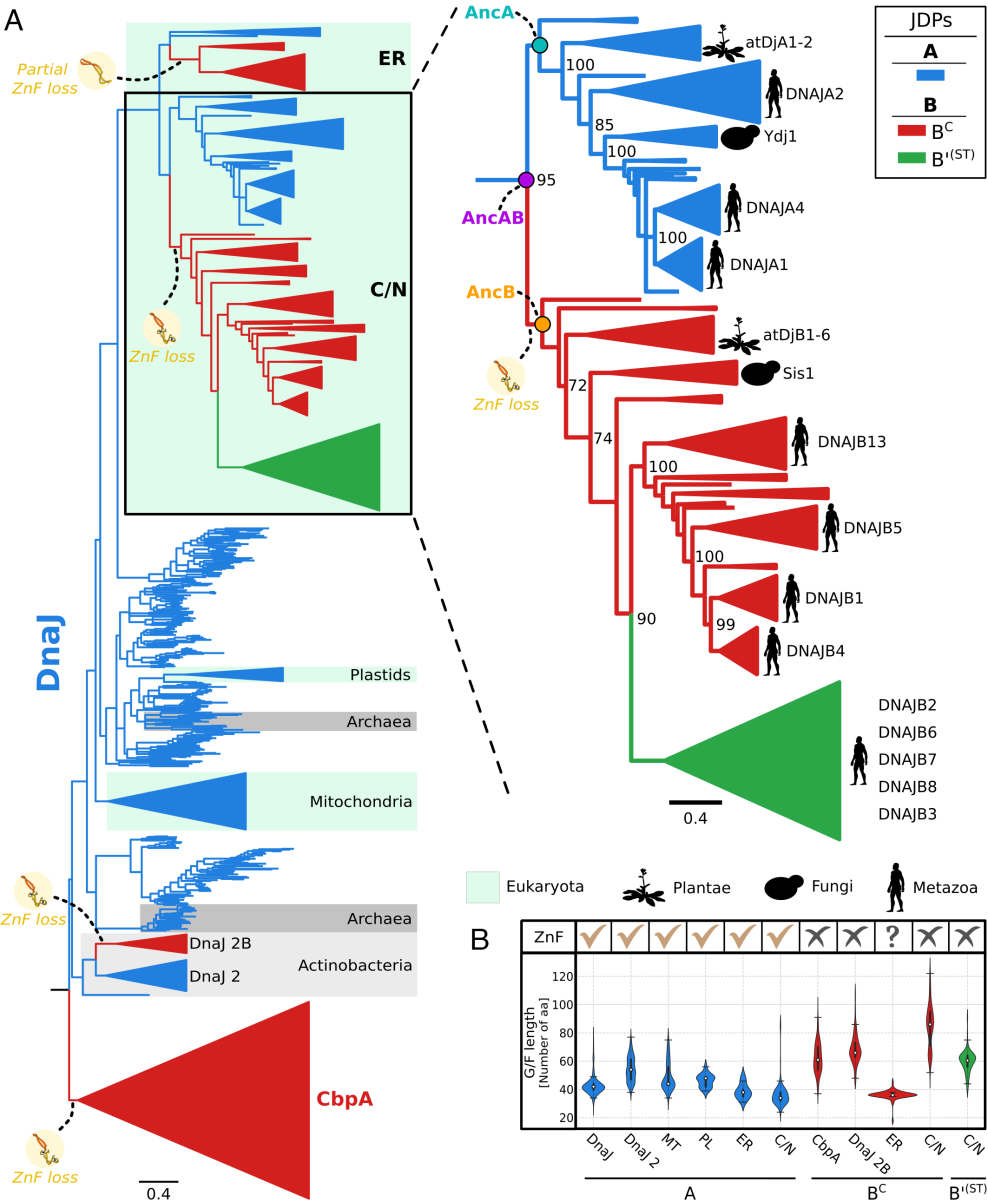
Evolutionary history of class A, B^C^, and B’^(ST)^ JDPs. (*A*) The best supported tree of class A (blue), B^C^ (red), and B’^(ST)^ (green) JDPs reconstructed with LG+I + G model (*SI Appendix*, Fig. S1). *Left*: JDPs highlighted: Archaea (dark gray); Actinobacteria (light gray, with additional JDP copies (DnaJ 2 and DnaJ 2B) indicated; eukaryotic subcellular compartments mitochondria, plastids, cytosol/nucleus—C/N, endoplasmic reticulum—ER (light green). Loss or partial loss of ZnF (yellow circles with graphic). *Right*: Zoom-in of JDPs of C/N. The phylogenetic position of representative members of class A, B^C^, and B’^(ST)^ JDPs from *Saccharomyces cerevisiae*, *Arabidopsis thaliana*, and *Homo sapiens* indicated. Circles represent the last common ancestors: class A and B^C^/B’^(ST)^ (AncAB, magenta); class A (AncA, cyan); class B^C^/B’^(ST)^ (AncB, orange). Scale bar—amino acid substitutions per position. Bootstrap support values for major splits are indicated. Splits with bootstrap < 50 were collapsed into polytomy. (*B*) Presence of ZnF and length of the G/F region. ZnF: present (✓), absent (χ), truncated (**?**). Violin plots represent the distribution of the number of residues constituting the G/F region. The median is marked with a white dot; black lines indicate the end of the first and the beginning of the third quartiles; the plot width is proportional to the number of sequences with a given number of residues. DnaJ—bacterial class A, DnaJ 2/DnaJ 2B—DnaJ class A/B^C^ homologs from Actinobacteria, MT—mitochondria, PL—plastids, ER—endoplasmic reticulum, C/N—cytosol/nucleus, CbpA—bacterial B^C^.

To resolve the evolutionary origin of class B JDPs, we carried out an extensive phylogenetic analysis using class A and B (B^C^ and B’^(ST)^) sequences retrieved from 747 bacterial, archaeal, and eukaryotic genomes. Of the 1,420 retrieved sequences, 710 had a full-length ZnF, thus forming the class A group, with the remaining, having no or a truncated ZnF, forming the class B^C^ (556 sequences) and class B’^(ST)^ (154 sequences) groups. We obtained well resolved and highly congruent phylogenetic trees with strong support for major JDP groups regardless of the method/model used or the presence/absence of B’^(ST)^ sequences in a dataset (Fig. 2 and *SI Appendix*, Figs. S1–S12). The trees revealed a complex phylogenetic distribution of class B^C^ JDPs. Not all members of this class cluster together as expected if B^C^s originated once in bacteria and were subsequently inherited by eukaryotic cells. Three B^C^ monophyletic groups branched off from class A JDPs, independently from the CbpA clade that contains most bacterial B^C^s–one in a subset of bacteria (Actinobacteria) and two in the eukaryotic lineage (Fig. 2).

**Fig. 3.**
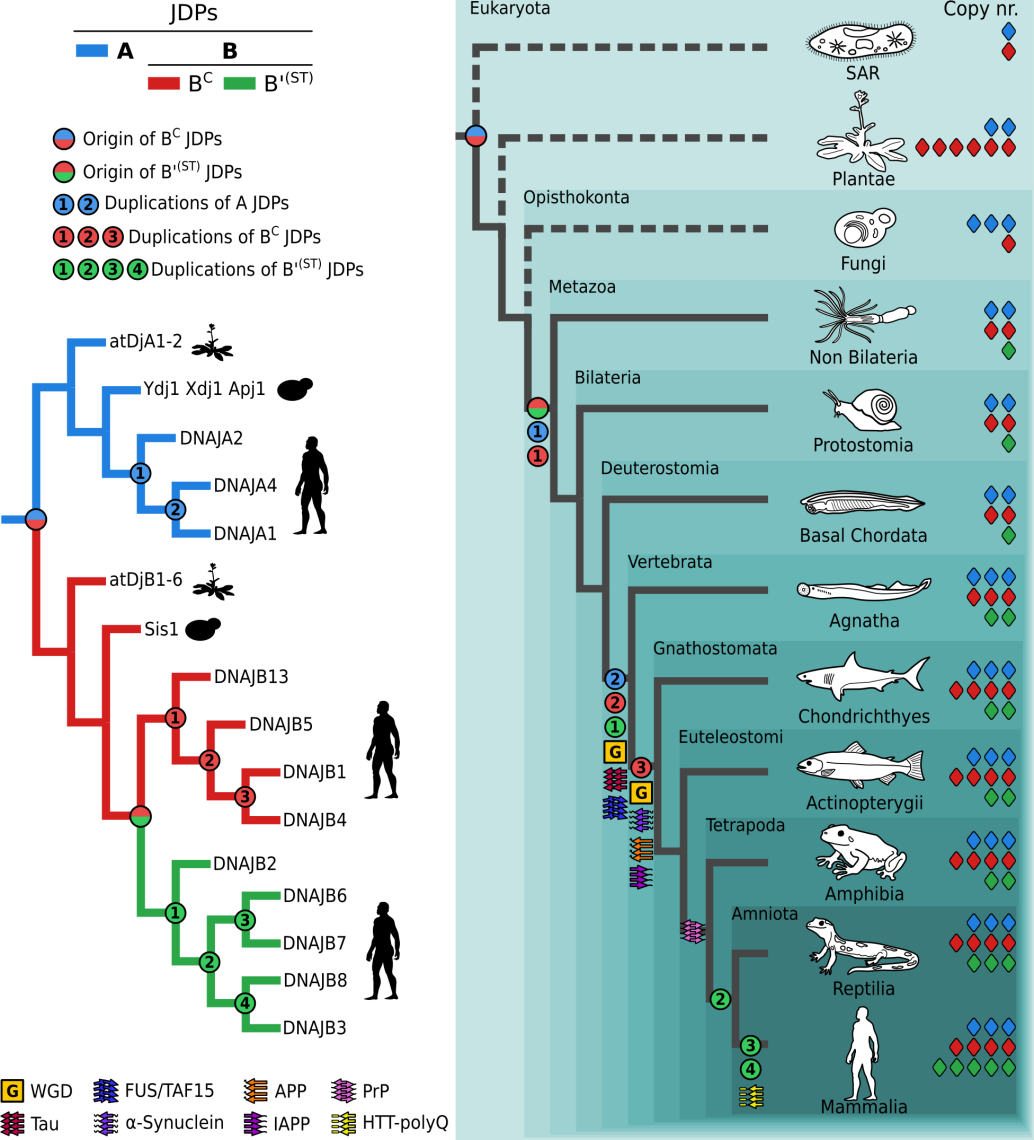
Evolutionary origin and expansion of the cytonuclear JDPs in the human lineage. *Left*: Simplified phylogeny of cytonuclear A (blue), B^C^ (red), and B’^(ST)^ (green) JDPs with names of *A. thaliana*, *S. cerevisiae,* and *H. sapiens*. *Right*: Placement of JDP gene duplication events and the emergence of human amyloidogenic precursor proteins on a simplified phylogeny of eukaryotes. Color coding of branches leading to JDP clades at *Left* and gene number at *Right* (diamonds): A (blue), B^C^ (red), B’^(ST)^ (green). Gene duplication events are marked with circles: blue/red—emergence of B^C^ from A ancestor; red/green—emergence of class B’^(ST)^ from B^C^ ancestor; blue (1, 2), sequential duplications of As; red (1, 2, 3), sequential duplications of B^C^s; green (1, 2, 3, 4), sequential duplications of B’^(ST)^s. *Bottom Left*: Key for the tree on *Right*—whole genome duplication events (G, orange square) and graphical symbols representing the emergence of predecessors of human amyloidogenic proteins. Dashed branches indicate nonmetazoan eukaryotes.

Our phylogenetic analysis traces the origin of uncharacterized B^C^ JDPs from Actinobacteria to a DnaJ duplicate (DnaJ 2) that emerged at the stem of this bacterial lineage, followed by complete ZnF loss in a subset of Actinobacteria. In the case of cytonuclear B^C^ JDPs, which also completely lack a ZnF, their origin was traced to two sequential class A gene duplications that took place in a common ancestor of all eukaryotes (Figs. 2 and 3). The first led to a common ancestor of all the A/B^C^s present in both the ER and the cytosol/nucleus. The subsequent duplication of the cytosol/nucleus class A gene led to sister cytonuclear class A and B^C^ JDPs. Most metazoans and plants have multiple B^C^s (e.g., four, DNAJB1, B4, B5, and B13, in humans and six, AtDjB1, B2, B3, B4, B5, and B6, in *A. thaliana*). In both, the multiple copies are more closely related to each other than to any class A or to B^C^ JDP from other lineages, including the single B^C^ present in fungi (e.g., Sis1 in *S. cerevisiae*). Such similarity indicates that multiplication of cytonuclear B^C^s was driven by lineage specific duplications of B^C^ genes. For example, three sequential duplications of B^C^ genes can explain the presence of the four B^C^ paralogs in humans (Figs. 2 and 3).

In sum, the branching pattern of class B^C^ JDPs indicates that each class B^C^ group shares common ancestry with a different class A JDP. More specifically, class B^C^ JDPs from the cytosol/nucleus of eukaryotic cells and from Actinobacteria are more closely related to their class A predecessor than to members of other B^C^ groups. These results imply that B^C^s independently evolved from class A ancestors via loss of the ZnF. That loss of the ZnF occurred independently, that is convergently, more than once raises the question of whether such loss enabled evolution of distinctive functional abilities, while substantially maintaining overall function. This idea is consistent with previous results (20, 21). For example, deletion of the ZnF’s β-hairpin (Fig. 1), while responsible for its unique interaction with oncogenic mutant of p53, does not affect interaction with other substrates (21). Thus, though the ZnF may have broadened substrate binding ability, both the innate rigidity of the ZnF (Fig. 1) and the inflexibility it imposes on the homodimer (33–35) may restrict conformational dynamics that are required for the unique functionality of B^C^s.

### Human Cytonuclear B’^(ST)^ JDPs Emerged From a B^C^ Ancestor.

We also conclude from our phylogenetic analysis that B’^(ST)^s originated from a cytonuclear B^C^ in the lineage leading to metazoans. On our best supported trees, B’^(ST)^s share a common ancestry with metazoan cytonuclear B^C^s and are only distantly related to fungal (Sis1) and plant (atDjB1-6) B^C^s (Fig. 2 and *SI Appendix*, Figs. S1–S3). Furthermore, the five human B’^(ST)^ paralogs (DNAJB2, B3, B6, B7, B8) are more closely related to each other than to any class B^C^ or A JDP, indicating they evolved by sequential duplications of B’^(ST)^ genes. We compared the distribution of B’^(ST)^s across metazoans with the branching order of their phylogeny to determine evolutionary relationships among them (*SI Appendix*, Fig. S13). We found that DNAJB2 branches off at the base of the B’^(ST)^ tree and is present in all metazoans, thus representing the predecessor of the B’^(ST)^s lineage (Fig. 3 and *SI Appendix*, Fig. S13). Its duplication in a common ancestor of vertebrates gave rise to the DNAJB6 lineage. DNAJB6’s subsequent duplication in a common ancestor of reptiles and mammals gave rise to the DNAJB8 lineage. Later, two independent duplications of DNAJB6 and DNAJB8 in a common ancestor of mammals gave rise to DNAJB7 and DNAJB3 respectively. Thus, four gene duplications can explain the presence of the five B’^(ST)^ JDPs in humans (Fig. 3). We also noted that the DNAJB2 duplication coincided with a whole genome duplication (WGD) event at the base of vertebrates (Vertebrata) (36). This and a subsequent WGD event at the base of jawed vertebrates (Gnathostomata) could have also been responsible for duplications of class A and B^C^ JDPs (Fig. 3). We also note that human DNAJB3 does not have S/T and βD due to a premature stop codon. The biological significance of this is not clear (37), as it is an exception. All DNAJB3 orthologs in our dataset, including chimpanzee—the species most closely related to humans (38)—lack a stop codon, and therefore have both S/T and βD.

The ancestry of S/T and βD domains of the B’^(ST)^s is not established by our analysis. These domains do not align with either B^C^ or A sequences. However, both βD and CTD1/2 of As and B^C^s are enriched in β-strands (Fig. 1). βD might have originated from CTD1/2 and then undergone major rearrangements, obscuring positional homology between the ancestral and daughter sequences (39). We therefore carried out analyses based on pair-wise comparisons of Hidden Markov model (HMM) sequence profiles, which capture conservation of each amino acid position in the sequence alignment, allowing detection of even weak homology (40). We prepared profiles of segments of βD and CTD1/2, as well as full J-domain and G/F region profiles of B’^(ST)^, B^C^, and A JDPs (*SI Appendix*, Fig. S14). Consistent with our phylogenetic results, the J-domain and G/F regions of B’s are more similar to those of B^C^s than those of As. However, no sequence homology between the βD and S/T profiles of B’^(ST)^s and those based on B^C^ and A sequences was detected, suggesting that the C-terminal domains of B’^(ST)^ did not evolve from B^C^ or A sequences. Therefore, B’^(ST)^s likely have a chimeric evolutionary origin. S/T and βD could have evolved via fusion with another gene (41). However, our B’^(ST)^ profiles did not detect any homologous sequences in prokaryotic or eukaryotic proteomes, nor did searches for βD type folds using Foldseek (42) reveal any structural homologs. Possibly, S/T and βD underwent such strong rearrangements during evolution that detectable homology with the source sequence/structure was lost (43) or they evolved de novo from noncoding DNA segment(s) (44).

Regardless of the origin of the S/T segment and βD domain, the phylogeny discussed above (Figs. 2 and 3) indicates that the J-domain and G/F region of B’^(ST)^s descended from a cytonuclear B^C^ predecessor. This origin is consistent with the shared features of the G/F regions. The G/F length of both cytonuclear B^C^s and B’^(ST)^s is longer than that of class A JDPs (Fig. 2*B*). Furthermore, the helical segment (helix V) (Fig. 1) within the G/F region of B’^(ST)^ interacts intramolecularly with its J-domain (45), regulating activity, as does helix V of class B^C^ JDPs (46, 47). We speculate that a longer G/F region facilitates the autoregulatory activity of helix V that is critical for the amyloid related activities of both B^C^s and B’^(ST)^s.

### Expansion of Cytonuclear B^C^ and B’^(ST)^ JDPs Predates Emergence of Pathogenic Amyloids in the Human Lineage.

The known ability of human B’^(ST)^ and B^C^ JDPs to engage with amyloids prompted us to investigate the relationship between their emergence/proliferation and the emergence of amyloidogenic proteins in the human lineage (Fig. 3). The only disease-causing amyloidogenic protein for which we found information about its emergence in the literature was the microtubule-associated protein (Tau) (48). We therefore reconstructed phylogenies of seven others for which there is experimental evidence that JDP/Hsp70 systems either affect their fibrilization or are able to dissociate their fibrils: Aβ protein precursor (APP), fused in sarcoma (FUS), TATA-box-binding protein-associated factor 15 (TAF15), islet amyloid polypeptide (IAPP), α-synuclein (α-syn), prion protein (PrP), huntingtin protein (HTT-polyQ) (*SI Appendix*, Figs. S15–S18). Our analyses revealed that Tau, as well as a FUS/TAF15 progenitor, emerged at the base of vertebrates (Vertebrata). Others emerged later: APP, IAPP, and α-syn at the base of jawed vertebrates (Gnathostomata); PrP at the base of four-legged vertebrates (Tetrapoda); and HTT-polyQ at the base of mammals (Mammalia) (Fig. 3). Thus, the emergence of all these amyloidogenic proteins was predated by both the emergence of B^C^ at the base of eukaryotes and the emergence of B’^(ST)^ at the base of metazoans. Subsequent duplications of both B’^(ST)^ and B^C^ coincided with the emergence of Tau and FUS/TAF15 precursor, while the final duplication of B^C^ coincided with the emergence of APP, IAPP, and α-syn (Fig. 3). The three duplications of B’^(ST)^ that took place at the base of amniotes (Amniota) (one duplication) and Mammalia (two duplications) roughly coincided with the emergence of PrP and HTT-polyQ.

Taken together, our results indicate that the B′^(ST)^ subfamily arose through a B^C^- gene duplication before the appearance of the disease-causing amyloids we examined, while the subsequent expansion of cytonuclear B′^(ST)^ and B^C^ paralogs in the human lineage coincided with their emergence. Thus, JDPs that can both prevent formation and facilitate disassembly of amyloid fibrils not only were in place before emergence of present-day disease-causing amyloid proteins in the human lineage, but they also multiplied in their presence.

### Ancestral Cytonuclear B^C^ Is Functionally Similar to Contemporary B^C^s and Different From Both Ancestral and Contemporary Class A JDPs.

The engagement of cytonuclear B^C^s, as well as the B′^(ST)^s that emerged from them, with amyloids motivated us to ask whether such functional properties emerged before or after ZnF loss. We resurrected ancestral proteins (49) from before (AncAB) and after (AncB and AncA) the class A gene duplication that led to emergence of B^C^s (Fig. 2). AncB has a domain composition typical of present-day B^C^s, including the lack of the ZnF, whereas AncAB and AncA have all the domains present in contemporary class A JDPs, including a full-length ZnF (*SI Appendix*, Fig. S19).

**Fig. 4.**
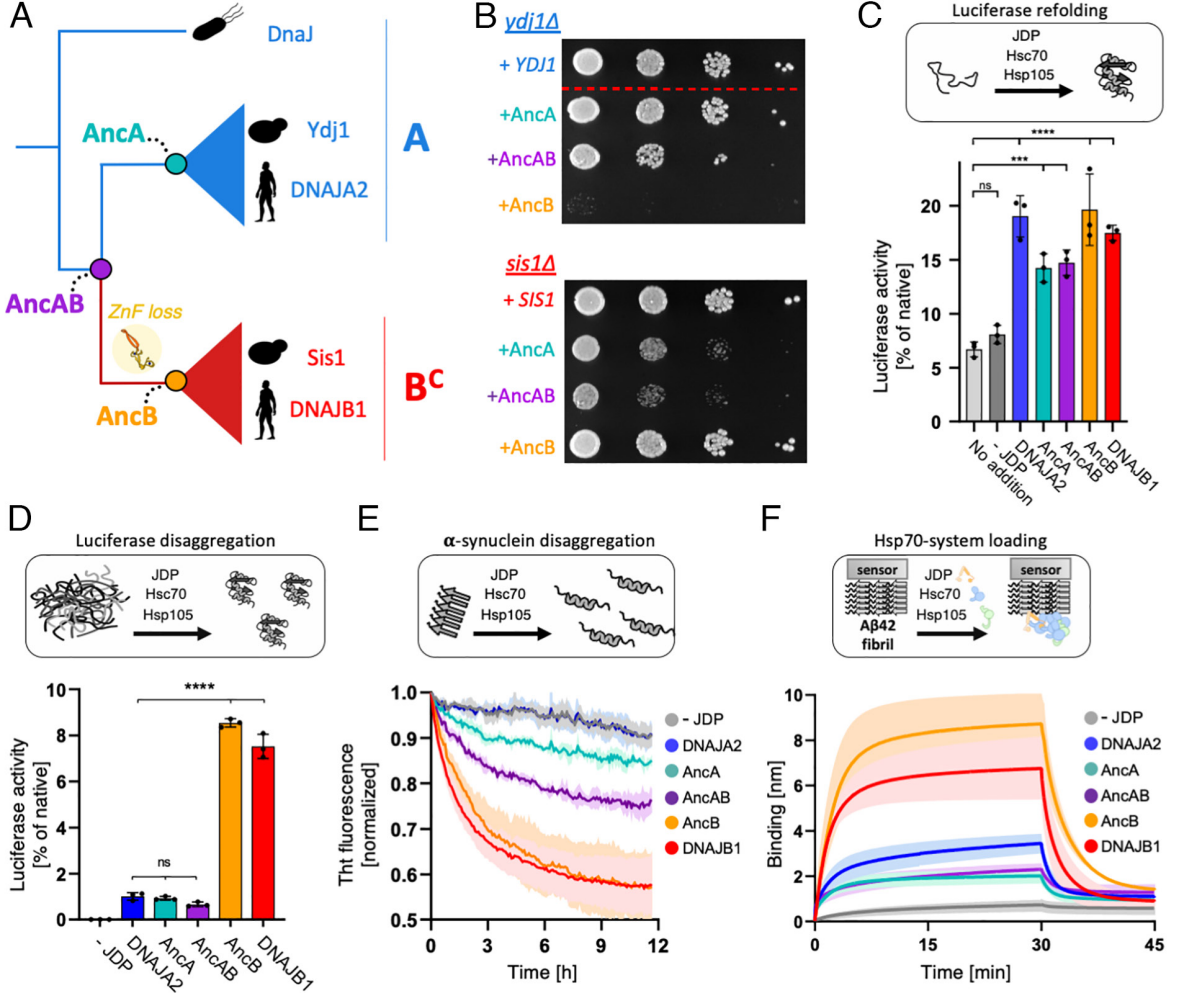
Functional divergence of B^C^ JDPs from the cytosol/nucleus. (*A*) B^C^s of the C/N share common ancestor (AncAB) with class A JDPs. Following AncAB gene duplication canonical Bs (B^C^) diverged from class A JDPs losing a ZnF domain, indicated on the branch leading to AncB. Sequences representing the common ancestors of B^C^s (AncB) and As (AncA), as well as AncAB, were reconstructed for use in subsequent experiments (*B*–*F*). (*B*) Ancestral JDPs are functional in vivo in yeast. *ydj1*Δ (*Top*) and *sis1*Δ (*Bottom*) cells harboring plasmid-borne copies of ancestral JDPs, WT *YDJ1,* or WT *SIS1*, as indicated, were plated as 10-fold serial dilutions on glucose-rich medium and incubated at 30 °C for 2 d. The dotted line indicates removal of nonrelevant sample from image. (*C*) Purified ancestral JDPs are active in chaperone-assisted refolding of Firefly Luciferase (FLuc). FLuc refolding at 100 nM was assayed upon dilution from 5 M guanidinium-HCl into buffer either without chaperones (spontaneous refolding) or with chaperones: Hsc70 at 3 μM, Hsp105 at 0.3 μM and the indicated contemporary or ancestral JDPs at 1 μM. (*D*) AncB, but neither AncAB nor AncA, has limited but measurable activity in FLuc disaggregation. Disaggregation of 200 nM FLuc aggregates in the presence of Hsc70 at 1.5 µM, Hsp105 at 0.15 µM and JDPs at 1 µM (*C* and *D*). FLuc activity was normalized to that of native FLuc. One-way ANOVA followed by Dunnett’s test versus control: ***P < 0.001; ****P < 0.0001; ns, not significant. Average activity of at least 3 replicates ± SD is shown. (*E*) AncB is more active than either AncAB or AncA in α-synuclein fibrils disassembly. Disassembly of α-synuclein fibrils in the presence of Hsc70 at 3 µM, Hsp105 at 0.3 µM, and indicated JDPs at 0.25 µM. Fibril disassembly was monitored by fluorescence of ThT, an amyloid-specific dye that reports on the total amyloid load in the sample. (*F*) AncB drives interaction of Hsc70 and Hsp105 with Aβ42 amyloid fibrils more efficiently than either AncAB or AncA. Interaction of Hsc70 at 1 µM and Hsp105 at 0.1 µM with Aβ42 amyloid fibrils immobilized on a BLI biosensor was monitored in the absence or presence of JDPs at 1 µM. (*E* and *F*) The lines represent the average of three replicates, with shading indicating SD. For statistical analysis of differences in JDP activities in panels *C*–*F*, see *SI Appendix*, Fig. S20*B* and S22.

As an initial step, we assessed whether these ancestral JDPs have any chaperone activity by testing their ability to substitute for present-day A and B^C^ in in vivo growth and biochemical protein refolding assays, using the budding yeast and human systems, respectively. Yeast strains having either a deletion of the chromosomal gene encoding B^C^ Sis1 or A Ydj1 (Fig. 4*B* and *SI Appendix*, Fig. S20*A*) were used (50, 51). Consistent with expectations—AncB supported robust growth of *sis1*∆ cells, but not *ydj1*∆; AncA supported robust growth of *ydj1*∆, but only minimal growth of *sis1*∆. AncAB did not support robust growth of either strain, albeit improved growth of *ydj1*∆ substantially more than *sis1*∆. We also tested the ability of purified ancestral proteins to refold an unfolded substrate protein, luciferase. Both human class A (DNAJA2) and B^C^ (DNAJB1) have been shown, in conjunction with human Hsc70 and NEF Hsp105, to be functional in this assay (22, 25). The three ancestral JDPs also had substantial activity (Fig. 4*C* and *SI Appendix*, Fig. S20*B*). Based on these in vivo and in vitro results, we concluded that these ancestral JDPs possess chaperone activities, allowing us to productively compare their functional properties with respect to protein aggregates.

Next, we tested ancestral JDPs in biochemical assays in which present-day canonical B^C^s are more active than As: (i) disaggregation of amorphous aggregates, (ii) disassembly of amyloid fibrils, (iii) recruitment of Hsp70/NEF to amyloid fibrils. In disaggregation assays of preformed luciferase aggregates carried out in the presence of Hsp70/NEF, AncB had low but measurable activity, as is typical of present-day B^C^s [human DNAJB1 (25) or yeast Sis1 (52)] (Fig. 4*D* and *SI Appendix*, Figs. S21 and S22). AncA and AncAB, like human DNAJA2, had marginal, barely measurable, activity. We observed a similar pattern in the assay, in which disassembly of preformed α-synuclein fibrils (*SI Appendix*, Fig. S23*A*) is assessed in the presence of ancestral proteins and human Hsp70/NEF (Fig. 4*E* and *SI Appendix*, Figs. S22 and S24). AncB was more active than either AncAB or AncA. The recruitment assay measured the ability of JDPs to facilitate interaction of Hsp70/NEF with preformed Aβ42 amyloid fibrils immobilized to an optical sensor (*SI Appendix*, Fig. S23*B*). AncB drove fast binding kinetics and high binding yield, similar to DNAJB1, while binding signals for both AncA and AncAB were low, comparable to DNAJA2 (Fig. 4*F* and *SI Appendix*, Figs. S22*C* and S25). We note that AncB is more active than contemporary DNAJB1 in both luciferase disaggregation and Hsp70/NEF recruitment onto Aβ42 amyloid (Fig. 4 *D* and *F* and *SI Appendix*, Figs. S20 and S22). Replacement of present-day Hsp70s/NEFs with their ancestors in future studies may help to elucidate the basis of this difference.

Our experimental analysis of ancestral JDPs indicate that AncB is more efficient than AncAB and AncA in both recruiting Hsp70/NEF chaperones to protein aggregates and in facilitating their disassembly (Fig. 4 *D*–*F*). Therefore, we conclude that these functionally important differences, which distinguish present-day cytonuclear B^C^s from their class A counterparts, evolved early on the branch connecting AncAB to the last common ancestor of the B^C^ lineage (AncB) (Fig. 4*A*). They are also consistent with our hypothesis that AncB diverged functionally after its emergence through a class A gene duplication, but subsequent to ZnF loss.

### Class B^C^ JDPs of the ER Emerged From Class A By Partial Loss of the ZnF.

**Fig. 5.**
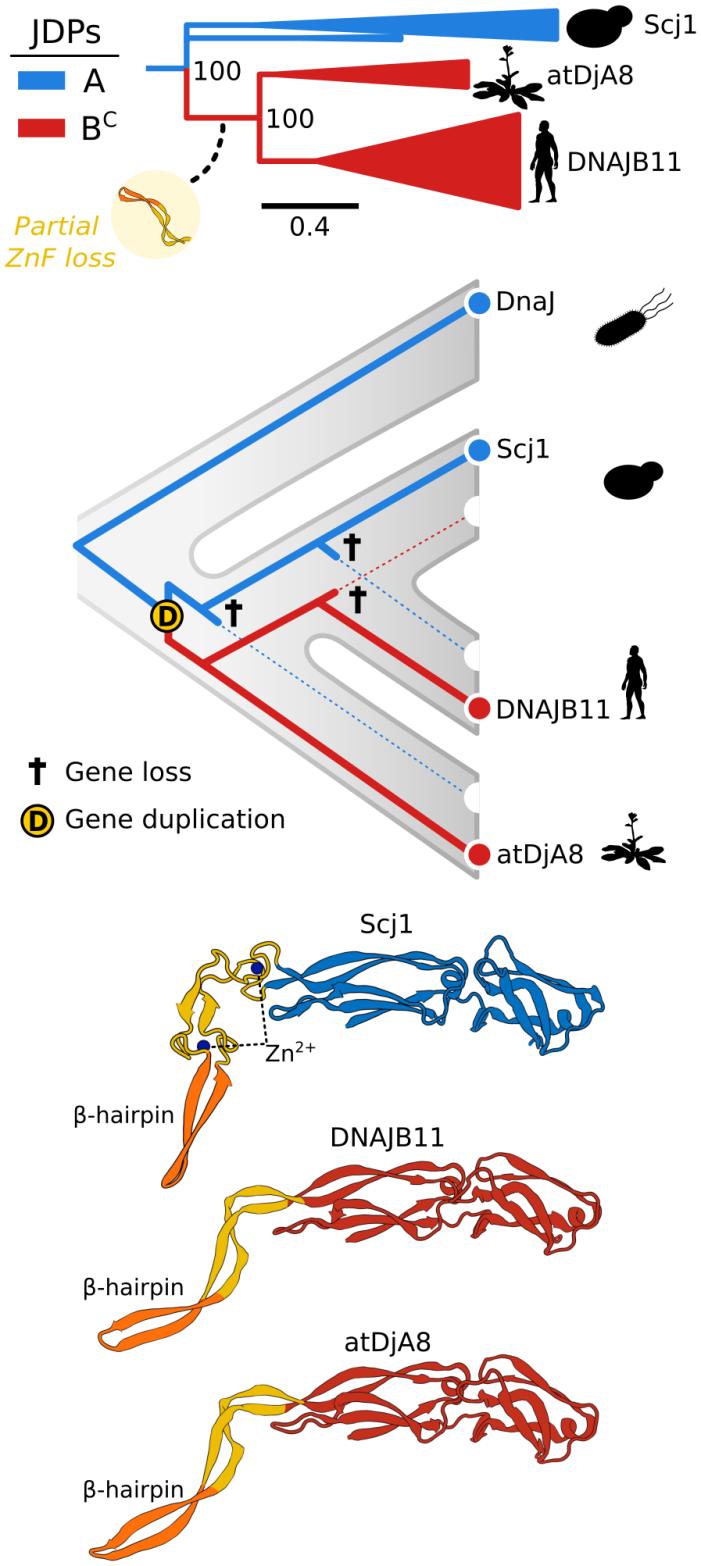
Evolution of class A and B^C^ JDPs from the ER. *Top*: Phylogeny of class A and B^C^ JDPs from the ER—class A (blue) and B^C^ (red) with names of *A. thaliana*, *S. cerevisiae,* and *H. sapiens* JDPs. Clades containing metazoans, fungi, and plants are collapsed into triangles. Partial loss of ZnF (yellow circle with graphic). Scale bar—amino acid substitutions per position. Bootstrap support for major splits is indicated. *Middle*: Scenario that could explain the emergence of B^C^s with a shorter ZnF, and the evolutionary relationships among class A (blue) and B^C^ (red) JDPs from the ER, presented in the context of species phylogeny (gray). Orange circle with “D” marks a duplication event prior to ZnF shortening (red line); cross (†) indicates the loss of a gene copy in a particular evolutionary lineage. *Bottom*: AlphaFold structural models of three contemporary JDPs of the ER: yeast Scj1 (ID: AF-P25303-F1), human DNAJB11 (ID: AF- Q9UBS4-F1) *A. thaliana* atDjA8 (ID: AF-Q9LZK5-F1). Full-length ZnF of Scj1 and truncated ZnF of DNAJB11 and atDjA8 (yellow); ZnF β-hairpin (orange).

The evolutionary relationships, as well as the basic classification, of class A/B^C^ JDPs of the ER have been debated for years based on data from model systems. Fungi (i.e., Scj1 in *S. cerevisiae*) have a class A JDP; metazoans and plants (i.e., DNAB11 and atDjA8 in human and *Arabidopsis thaliana*, respectively) have a JDP with a truncated ZnF - 30 rather than the 60 residues (with 4, not 8, cysteines) found in full-length class A ZnFs (17, 53, 54). Results of the analysis of our dataset confirmed this distribution—a broad presence of full-length ZnF in fungi and unicellular eukaryotes, and a truncated ZnF in metazoans and plants (*SI Appendix*, Fig. S26). But unexpectedly, all belong to a single clade (Figs. 2 and 5)—with fungal As only distantly related to metazoan and plant B^C^s—inconsistent with the well-established sister relationship of metazoans and fungi, and their distant relationship to plants (Fig. 5) (55). The emergence of class B^C^ JDPs after a class A gene duplication in a common ancestor of metazoans, fungi, and plants (Fig. 5) could explain our observed relatedness between ER JDPs—if one duplicate subsequently evolved a truncated ZnF (class B^C^), while the other maintained a full-length ZnF (class A), followed by differential gene loss (Fig. 5).

The longstanding discussion about JDPs of the ER has focused on whether the B^C^s of plant/metazoan are functionally divergent from the As of fungi (15, 56). For our analysis, we followed the strict definition of class A JDPs (18) and placed those with a truncated ZnF in class B^C^. However, similar structures are predicted for full-length and truncated ZnF domains (54) with the β-hairpin critical for substrate interaction (21) similarly protruding (Fig. 5). Considering the oxidative environment of the ER, a short ZnF stabilized by disulfide bonds (53) could result in a structure similar to that of zinc coordinated full-length ZnF of *E. coli* DnaJ and cytosolic *S. cerevisiae* Ydj1 (33, 34), thereby maintaining class A functionality.

The length of the G/F region of B^C^s of the ER is also consistent with class A-like functionality (Fig. 2*B*). Their 36-residue median length is very similar to that of As of both the ER and cytosol/nucleus (38 and 34 residues, respectively), while that of cytonuclear B^C^ JDPs is significantly longer (86 residues). The greater G/F length of cytonuclear B^C^s is likely attributable in part to the specialized inhibitory mechanism that has been found in both budding yeast and human B^C^s—the helical segment (helix V) in the G/F folds back on the J-domain regulating its activity (46, 47). Furthermore, B’^(ST)^s, which have a median G/F length of 60 residues have been shown to have a similar intramolecular regulatory mechanism (45). Although a conserved G/F helical segment is present in the vast majority of class A and B JDPs (47) (*SI Appendix*, Fig. S27*A*), sequence comparison revealed a residue composition both within and upstream of the inhibitory helices of cytonuclear B^C^s and B’^(ST)^s that is not found in either cytonuclear As or A/B^C^s of the ER (*SI Appendix*, Fig. S27*B*).

The short G/F regions and sequence composition of their helices are consistent with the idea that B^C^s from the ER did not functionally diverge from their class A predecessors. But when considering class B JDPs more broadly, it is interesting to note that, though the differences are not as great, the G/Fs of CbpA and DnaJ 2B of Actinobacteria are also longer (on average) than those of class A JDPs (Fig. 2*B*). The longer G/F region of these bacterial B JDPs and the cytonuclear B JDPs must have evolved convergently, as they independently originated from class A genes, implying functional importance. Future experimental comparisons of class B^C^ JDPs from different evolving lineages may provide insight into their functional diversity.

## Conclusions

Broad taxonomic distribution (15, 16) of class A and B^C^ JDPs has long been assumed to reflect their deep evolutionary origin at the stem of the bacteria lineage. But our detailed phylogenetic analyses revealed that class B^C^ JDPs emerged independently more than once from class A ancestors—in bacteria and in eukaryotes. Nevertheless, our findings do lend support to the traditional, DnaJ centered, classification of JDPs (18). Both our results and recent surveys of JDP diversity (15, 16) indicate that class A members have the broadest phylogenetic distribution. Thus, DnaJs are likely the progenitor of all JDPs, even those sharing only a J-domain with class A and B members [i.e., class C (5)], receiving their J-domains from DnaJ homologs, as recently proposed for the class C JDP that function in the biogenesis of iron-sulfur clusters, HscB (57).

That loss of the ZnF in a class A progenitor occurred independently, that is convergently, in bacteria and eukaryotes suggests that this structural simplification allowed acquisition of new functions and can be considered an example of reductive evolution at the protein level. Such domain losses during evolution of eukaryotic protein families are common (58) and thought to have contributed to accelerated evolution of novel gene paralogs (59–61). Once in place, both B^C^s and B’^(ST)^s duplicated several times. The increase in gene number of both B^C^ and B’^(ST)^s is consistent with a recent proposal that the demand for chaperone activity upon expansion of the proteome across the tree of life (62) was often fulfilled not by the emergence of new chaperone families but by an expanded network of cochaperones, including JDPs (63). We speculate that there is such a causal relationship between multiplication of pathogenic amyloids and the presence of class B JDPs in the cytosol/nucleus of human lineage.

## Materials and Methods

### Dataset of Class A and Class B JDPs and Evolutionary Analyses.

To resolve evolutionary relationships among A, B^C^, and B’^(ST)^, JDPs, we assembled a dataset of 747 proteomes from bacteria, archaea, plants, fungi, metazoans, and protists. We retrieved JDP sequences using Hidden Markov model (HMM) sequence profiles. We used Hmmer1.3b1. to generate two profiles based on the well-characterized class A and B^C^ sequences from model systems (*E. coli*, *A. thaliana*, *S. cerevisiae,* and *H. sapiens*) aligned with Clustal Omega v1.2.2. The first profile encompassed J-domains and G/F regions (JD-GF-HMM)—diagnostic features of class A and B^C^/B’ JDPs. The second profile encompassed C-terminal domains (CTD-DD-HMM)—diagnostic feature of class A and B^C^, but not B’. Sequences retrieved by both profiles that also had a ZnF were assigned to class A, those that do not have a full-length ZnF, were assigned to class B^C^. Sequences retrieved only by JD-GF-HMM but not by CTD-DD-HMM were assigned as class B’. Class A and B^C^ sequences were aligned and curated manually—incomplete or unusually fast evolving sequences were removed. Following realignment of curated A and B^C^ sequences, a full-length HMM profile was built (AB^C^-HMM). This profile was used to prepare two alignments of A/B^C^ sequences for phylogenetic analyses: one for all A/B^C^ sequences from our dataset (AB^C^-alignment); the other restricted to eukaryotic A/B^C^ sequences (AB^C^E-alignment). To resolve the evolutionary origin of cytonuclear B’^(ST)^ JDPs of metazoans, whose diagnostic features are a S/T segment followed by a βD domain, we retrieved B’^(ST)^ sequences from our dataset of B’ JDPs using the B’^(ST)^-HMM profile based on the alignment of four human B’^(ST)^s: DNAJB2, B6, B7, and B8. For phylogenetic analyses these sequences were aligned to the AB^C^-HMM profile along with class A and B^C^ sequences; AB^C^B’^(ST)^-alignment. To analyze evolutionary relationships among B’^(ST)^, the B’^(ST)^ dataset was completed by addition of sequences from Agnatha and basal Chordata retrieved from KEGG and OMA databases. Next the incomplete or unusually fast evolving sequences were removed resulting in B’^(ST)^-alignment of 126 sequences. For each position of the AB^C^, AB^C^E, AB^C^B’^(ST)^, and B’^(ST)^-alignments, posterior probability, which represents a degree of confidence in each position (residue or gap), was computed using Forward–backward algorithm (64). Positions with posterior probability >0.5 were used for phylogenetic tree reconstructions (65). For phylogenetic methods and other evolutionary analyses see Supporting Information.

### Identification of Molecular Features of Class A, B^C^, and B’^(ST)^ JDPs.

The presence of a ZnF was recognized based on the conserved sequence block in the AB^C^B’^(ST)^-alignment corresponding to the ZnF of bacterial DnaJ, and the presence within it of 8 cysteine residues positioned such that they could coordinate two zinc ions. The length of the G/F region calculated using the AB^C^B’^(ST)^-alignment, corresponds to the number of amino acids within the conserved sequence block matching the G/F region of bacterial DnaJ. The presence of helix V in the G/F region was identified as described in (47) using AB^C^B’^(ST)^-alignment and AlphaFold structural models of analyzed JDPs. Helix V was recognized when two criteria were fulfilled: i) a helical secondary structure was inferred within the G/F region using DSSP software (66); ii) this helical structure corresponded to the conserved sequence block in the AB^C^B’^(ST)^-alignment that matches the helix V residues of DNAJB1, DNAJB6, and Sis1. Alignment logos were generated using the WebLogo server (67).

### In Vivo Assay in Yeast.

To test in vivo function of AncA, AncB, and AncAB *S. cerevisiae* haploid strains isogenic to the W303 background and carrying a deletion of the chromosomal copy of *SIS1* or *YDJ1* (i.e., *sis1*Δ or *ydj1*Δ) were used. *sis1*Δ and *ydj1*Δ carrying a *URA3*-based plasmid having a wild-type (WT) copy of Sis1 [YCp50-*SIS1*(50)] or Ydj1 [pRS316-*YDJ1* (51)], respectively, were transformed with a plasmid encoding a test protein or, as controls, WT Sis1, or WT Ydj1, and then plated on complete minimal media plates containing 5 fluoroorotic acid (5-FOA) (TorontoResearch Chemicals Inc., Canada) (68) to select for cells that had lost the *URA3* plasmid containing the WT gene. Growth assays of the resulting strains were performed by serially diluting cells in 10 -fold increments and spotting on rich media plates [YPD, (1% yeast extract, 2% peptone) (Difco Laboratories, Detroit, MI), 2% dextrose] starting with 5,000 cells in the first position.

### Purification of Proteins Used in Biochemical Assays.

Published protocols were used for purification of DNAJA2, DNAJB1, Hsc70, Hsp105 (25), Sis1 (69), Ydj1 (70) Ssa1, Sse1 (71), and His-tagged luciferase (72). AncA, AncB, and AncAB were purified using the protocol for DNAJB1(25). α-synuclein was expressed from the pET21a-α-synuclein plasmid (a gift from prof. Bernd Bukau, University of Heidelberg) in BL21(DE3) cells at 37 °C. After cell lysis in a buffer containing 10 mM Tris-HCl pH 7.5 and 1 mM EDTA, the lysate was loaded onto Q-Sepharose (GE Healthcare) and eluted with a NaCl gradient up to 750 mM. The protein solution was dialyzed against 5 mM KPi, pH 7.4, and then loaded onto Hydroxyapatite (BioRad) and eluted with a gradient of 5-450 mM KPi, pH 7.4, followed by gel filtration on a Superdex 20 column (GE Healthcare) in buffer containing 20 mM HEPES-KOH pH 7.4. Finally, the protein was filtered through a 50,000 MWCO centrifugal filter (Millipore) to remove the high molecular mass impurities.

### Amyloid Fibrils Formation and Imagining.

α-synuclein fibrils were prepared essentially as described (73). 200 μM α-synuclein monomers were incubated for 15 d in buffer (20 mM HEPES-KOH pH 7.4, 100 mM NaCl) at 37 °C, with shaking at 1,000 rpm. Fibrils were separated from monomers by centrifugation (20,000×*g*, 30 min). The fibrils were resuspended in 20 mM HEPES-KOH pH 7.4, sonicated and stored at −80 °C. Fibril formation was confirmed using Thioflavin T (ThT) fluorescence and atomic force microscopy (AFM) imaging (*SI Appendix*, Fig. S23).

Aβ42 and C-terminal biotin-labeled Aβ42 peptides were synthesized via solid-phase peptide synthesis. Peptides were dissolved, and the monomeric fraction isolated as described (74). To induce fibrillation biotin-Aβ42 and Aβ42 peptides (10 µM) were mixed at a 1:10 ratio in buffer (50 mM Tris-HCl (pH 7.5), 150 mM KCl, 15 mM MgCl_2_) and incubated at 37 °C for 1 h with shaking at 750 rpm. Aβ42 amyloid fibril formation was confirmed using AFM imaging (*SI Appendix*, Fig. S23).

AFM visualizations of Aβ42 and α-synuclein fibrils were carried out using BioScope Resolve (Bruker, Bremen, Germany) at 23 °C in air. Each sample was deposited onto a freshly cleaned mica surface and incubated for 5 min at room temperature, followed by washing with deionized water and drying with streams of nitrogen gas prior to imaging using the ScanAsyst-Fluid+ probe (Bruker) (resonant frequency f0 = 150 kHz; spring constant k = 0.7 N/m). Images were taken at 512 × 512 pixels with a PeakForce Tapping frequency of 1 kHz and an amplitude of 150 nm. Height sensor signal was used to display the protein image using NanoScope Analysis v1.9 (Bruker, Bremen, Germany).

### Luciferase Refolding and Disaggregation.

Firefly luciferase (FLuc) refolding was carried out as described (75). FLuc (Promega) (20 μM) was chemically denatured in 5 M guanidine hydrochloride (GuHCl) and 10 mM dithiothreitol (DTT) for 1 h at 25 °C. The refolding reaction was initiated by 200-fold dilution into buffer (25 mM HEPES-KOH pH 7.5, 100 mM KCl, 10 mM Mg(OAc)_2_, 2 mM DTT, 5 mM ATP, 0.05% Tween20) either without or with chaperones: Hsc70, 3 μM; Hsp105, 0.3 μM, and JDP, 1 μM (DNAJB1, AncB, AncAB, AncA, or DNAJA2).

FLuc disaggregation was carried out as described (52). FLuc (20 μM) was incubated in buffer (25 mM HEPES-KOH pH 7.5, 75 mM KCl, 15 mM MgCl_2_) with 6 M urea at 25 °C for 10 min. It was then transferred to 48 °C for 10 min and subsequently diluted 25-fold into buffer without urea. After 5 min of incubation at 25 °C, the disaggregation reactions were initiated by the adding chaperones at the following concentrations: Hsc70, 1.5 μM; Hsp105, 0.15 μM, and JDP, 1 μM. FLuc activity was measured using the Luciferase Assay Kit (E1501, Promega) with a GloMax® 20/20 Luminometer (Promega).

Statistical analyses of chaperone activities were performed using one-way ANOVA implemented in GraphPad Prism Version 8.0.1. with Dunnett or Tukey post hoc tests.

### α-Synuclein Disaggregation Assay.

Disaggregation of α-synuclein fibrils by chaperones was monitored using changes in ThT fluorescence over a 12-h period as described (73). Reaction mixtures were prepared in a 96-well half-area nonbinding microplate (Corning 3881). Preformed α-synuclein fibrils (0.8 μM) were incubated at 30 °C in buffer (25 mM HEPES-KOH (pH 7.5), 75 mM KCl, 15 mM MgCl_2,_ 2 mM DTT, 2 mM ATP), ATP regeneration system (8 mM phosphoenolpyrufic acid (PEP) and 20 ng/μl pyruvate kinase) and 30 μM ThT with Hsc70, 3 μM; Hsp105, 0.3 μM, and JDP (DNAJB1, AncB, AncAB, AncA, DNAJA2), at the indicated concentrations. ThT fluorescence was measured using Tecan Spark plate-reader (excitation: 440 nm, emission: 480 nm). Background ThT fluorescence from buffer and chaperones was subtracted, and all intensities were normalized to the fluorescence at *t* = 0 min. The data shown represent the average of three independent experiments, with shaded areas indicating the SD.

### Recruitment of Hsc70/Hsp105 to the Sensor Immobilized Aβ42 Amyloid Fibrils.

Biotin-labeled Aβ42 fibrils were immobilized onto the streptavidin sensors (Octet^®^ SA Biosensor) for 15 min in BLI buffer (50 mM Tris-HCl, pH 7.5; 150 mM KCl; 15 mM MgCl_2_). Prior to recruitment measurements, the fibrillar nature of the immobilized Aβ42 was confirmed using anti-amyloid antibodies (Sigma-Aldrich, AB2287) at 1:100 dilution (*SI Appendix*, Fig. S23). To measure chaperone recruitment, the sensors with immobilized Aβ42 fibrils were immersed for 1,800 s in solutions containing BLI buffer supplemented with 2 mM DTT and 5 mM ATP and the following chaperones: Hsc70, 1 µM; Hsp105, 0.1 µM; and JDP, 1 µM (DNAJB1, AncB, AncAB, AncA, or DNAJA2). To measure chaperone dissociation, the sensors were subsequently immersed for 900 s in protein-free solution. Experiments were conducted using BLItz (Pall ForteBio) and Octet K2 (Satorius) instruments at room temperature.

To quantify the levels of Hsc70 interacting with Aβ42 amyloid fibrils, Hsc70 labeled with the Alexa Fluor 488 fluorescent dye (A488*-Hsc70) was used (*SI Appendix*, Fig. S25*B*). A sensor with immobilized Aβ42 fibrils was incubated for 30 min with a mixture of A488*-Hsc70 (1 µM), Hsp105 (0.1 µM), and ancestral or contemporary JDP (1 µM) in BLI buffer (association step). Fluorescence was measured after the sensor was incubated for 15 min in BLI buffer without proteins (dissociation step).

## Supplementary Material

Appendix 01 (PDF)

Dataset S01 (XLSX)

Dataset S02 (TXT)

Dataset S03 (TXT)

Dataset S04 (TXT)

Dataset S05 (TXT)

Dataset S06 (TXT)

Dataset S07 (TXT)

Dataset S08 (TXT)

Dataset S09 (TXT)

Dataset S10 (XLSX)

## Data Availability

All study data are included in the article and/or supporting information.
