# Supplementary material for: Origin of class B J-domain proteins involved in amyloid transactions
Source: Proc Natl Acad Sci U S A. 2026 Jan 9;123(2):e2522403123. doi: 10.1073/pnas.2522403123 (PMC12799103; doi:10.1073/pnas.2522403123)
Supplement: Supplementary file 1 — Appendix 01 (PDF) [file pnas.2522403123.sapp.pdf]

**Supplementary Methods**

**Phylogenetic tree reconstruction.**
Based on the AB$^{C}$ and AB$^{C}$B'$^{(ST)}$-alignments Maximum Likelihood (ML) trees were reconstructed. 1,000 ML searches (using IQ-TREE 2.3.6) (1) were conducted with 100 rapid bootstrap replicates using four method/model combinations: (i) LG model with proportion of invariable sites and gamma distribution (LG + I + G) - the best-fit model by Bayesian Information Criterion with ModelFinder (2), (ii) C10 and (iii) C20 heterogeneous mixture models (3) with the LG + G - to account for sequence composition bias, (iv) jackknife 60 method that produced majority-rule consensus tree based on ML trees reconstructed using 100 replicates assembled by sampling randomly 60% of positions from a sequence alignment - to account for position bias. Each of the four model/method combinations provided an alternative estimate of support for the major splits. The trees were rooted at the common ancestor of bacterial DnaJ (class A) and CbpA (class B$^{C}$). We chose this rooting, because their presence in all bacterial taxonomic groups, but very limited presence (DnaJ) or absence (CpbA) in archaea, implies common ancestry in bacteria. This rooting was statistically supported by several tests when compared to alternatively rooted topologies (Table S1).

For the AB$^{C}$E-alignment, phylogenetic trees were reconstructed using Bayesian approach with PhyloBayes 3.3e. We used four models of sequence evolution: (v) LG+I +G, (vi) WLSR5 (vii) C60+LG+G and (viii) CAT+LG+G. For each model two independent runs were performed for >4000 cycles, until the maxdiff was < 0.3 and the minimum effective size was >50. To construct the tree, the first 600 cycles were discarded as burn-in, and the topology and posterior consensus support was computed based on the trees from the remaining cycles.

For B'$^{(ST)}$-alignment the best fit JTT+I+G model was used. To obtain ML B'$^{(ST)}$ phylogeny concordant with the species phylogeny and phylogenetic distribution of B'$^{(ST)}$s across metazoans (suppl. Fig. S13A) the tree was constrained to enforce monophyly of a clade encompassing DNAJB8/B6/B3 homologs from mammals and reptiles (suppl. Fig. S13B). Constrained topology has a higher score than unconstrained topology (0.552 vs 0.448) using the approximately unbiased (AU) test (4). Pictures of representative members of taxonomic groups are original or modified pictures from PhyloPic (PhyloPic: A Library of Silhouettes for Phylogenetic and Taxonomic Research.).

**Ancestral protein resurrection.**
Ancestral (AncA, AncB and AncAB) sequences were reconstructed based on an AB$^{C}$-alignment and phylogeny (suppl. Fig. S5). Both the alignment and the tree were pruned to reduce the number of taxa but maintain species diversity in major JDP clades. Marginal reconstruction of ancestral sequences was performed with FastML using the empirical Bayes method with ML reconstruction of insertions and deletions (5). Ancestral amino acid sequences (suppl. Fig. S19) were converted into DNA sequences using codons optimal for expression in *E. coli*. These DNA sequences were cloned into plasmid pET-24a(+) for expression in *E. coli* and subsequently subcloned as BamHI-XhoI DNA fragments into similarly digested p414TEF plasmid for testing their *in vivo* functionality in *S. cerevisiae*.

**Pairwise profile-profile comparison between B'$^{(ST)}$ and A/B$^{C}$ domains.**
To compare similarity between domains of the B'$^{(ST)}$ JDPs and domains of A and B$^{C}$ JDPs we prepared segment-specific HMM profiles based on the A, B$^{C}$ and B'$^{(ST)}$ sequences from metazoans. Sequences were aligned and divided into segments corresponding to structural regions (suppl. Fig. S14). HMM profiles were generated for each of these segments using the hhmake tool from HH-suite v3.3.0. Segment-specific profiles were compared in pairs (B'$^{(ST)}$s profiles against profiles of either As or B$^{C}$s) using the hhalign tool from the HH-suite v3.3.0 to assess the sequence similarity between corresponding domains from B'$^{(ST)}$ and A or B$^{C}$ JDPs. To search for structural homologs of $\beta$D domain of B'$^{(ST)}$s we searched PDB100, AFDB, BFVD and SCOPe databases using Foldseek (6) with $\beta$D of DNAJB6 structure (PDB: 7JSQ) as a query.

**Phylogenetic analyses of amyloidogenic proteins.**

For phylogenetic analyses of amyloidogenic proteins (α-syn, APP, IAPP, FUS/TAF15, PrP and HTT-PolyQ), we prepared datasets of their amino acid sequences retrieved from the respective orthologous groups (HOG or OMA) available in the OMA database. To identify their homologs not captured by orthologous group assignment, the human amyloidogenic proteins listed above were used as query in BLASTP searches against representative proteomes from major metazoan lineages. For further validation, HMM profiles were prepared using HMMER v3.3.2 based on OMA-derived sequence sets for each protein family. These profiles were then used to search our dataset of 747 proteomes. In each case, sets were supplemented with additional sequences to provide representation of every major metazoan lineage. Final sets contained: 545 (APP), 59 (HTT), 348 (α-syn), 209 (PrP), 426 (FUS/TAF15), and 514 (IAPP) sequences. Sequences from each dataset were aligned with Clustal Omega v1.2.2. Poorly aligned positions were manually removed from the alignments. Phylogenetic trees were reconstructed using IQ-TREE 2.3.6. For each set 1,000 ML searches were conducted with 100 rapid bootstrap replicates. The best fit sequence evolution model was selected for each dataset (see suppl. Fig. S15-18). Using these phylogenetic trees, we traced the origin of each amyloidogenic protein to a common ancestor of a clade that includes its human precursor protein. For example, the origin of α-syn was traced to the common ancestor of jawed vertebrates (Gnathostomata) (suppl. Fig. S15B). In the case of HTT-PolyQ, its origin was traced to a common ancestor of mammals, as only mammalian HTTs have an expanded number of Q residues (suppl. Fig. S18).

**References**


1. B. Q. Minh *et al.*, IQ-TREE 2: New models and efficient methods for phylogenetic inference in the genomic era. *Mol. Biol. Evol.* **37**, 1530–1534 (2020).
2. S. Q. Le, O. Gascuel, An Improved General Amino Acid Replacement Matrix. *Mol. Biol. Evol.* **25**, 1307–1320 (2008).
3. H.-C. Wang, B. Q. Minh, E. Susko, A. J. Roger, Modeling Site Heterogeneity with Posterior Mean Site Frequency Profiles Accelerates Accurate Phylogenomic Estimation. *Syst. Biol.* **67**, 216–235 (2018).
4. H. Shimodaira, An Approximately Unbiased Test of Phylogenetic Tree Selection. *Syst. Biol.* **51**, 492–508 (2002).
5. T. Pupko, I. Pe, R. Shamir, D. Graur, A Fast Algorithm for Joint Reconstruction of Ancestral Amino Acid Sequences. *Mol. Biol. Evol.* **17**, 890–896 (2000).
6. M. v. Kempen *et al.*, Fast and accurate protein structure search with Foldseek. *Nat. Biotechnol.* **42**, 243–246 (2024).

**Table S1.** Likelihood values and statistical support for alternative rooting of the ML phylogeny of class A and $B^C$ JDPs.

| Outgroup | logL | ΔL | bp-RELL | p-KH | p-SH | c-ELW | p-AU |
|---|---|---|---|---|---|---|---|
| CbpA | -362375.91 | 0.00 | 0.0644 | 0.542 | 1 | 0.2 | 0.533 |
| DnaJ 2 | -362375.91 | $3.88*10^{-5}$ | 0.146 | 0.458 | 0.458 | 0.2 | 0.467 |
| DnaJ Archea | -362375.91 | $3.88*10^{-5}$ | 0.207 | 0.458 | 0.458 | 0.2 | 0.468 |
| CbpA+DnaJ 2 | -362375.91 | $3.88*10^{-5}$ | 0.262 | 0.458 | 0.458 | 0.2 | 0.334 |
| DnaJ+CbpA+DnaJ 2 | -362375.91 | $3.88*10^{-5}$ | 0.320 | 0.458 | 0.458 | 0.2 | 0.446 |

**logL**– log-likelihood value

**ΔL**– difference in log-likelihood compared to the best-fitting topology

**bp-RELL**– bootstrap proportion using RELL method (Kishino et al. 1990)

**p-KH**– p-value of one sided Kishino-Hasegawa test (1989)

**p-SH**– p-value of Shimodaira-Hasegawa test (2000)

**c-ELW**– Expected Likelihood Weight (Strimmer & Rambaut 2002)

**p-AU**– p-value of approximately unbiased (AU) test (Shimodaira, 2002)

---


- Kishino, H., Miyata, T., & Hasegawa, M. (1990). Maximum likelihood inference of protein phylogeny and the origin of chloroplasts. *Journal of Molecular Evolution*, *31*(2), 151-160.
- Kishino, H., & Hasegawa, M. (1989). Evaluation of the maximum likelihood estimate of the evolutionary tree topologies from DNA sequence data, and the branching order in Hominoidea. *Journal of molecular evolution*, *29*(2), 170-179.
- Shimodaira, H., & Hasegawa, M. (1999). Multiple comparisons of log-likelihoods with applications to phylogenetic inference. *Molecular biology and evolution*, *16*(8), 1114.
- Strimmer, K., & Rambaut, A. (2002). Inferring confidence sets of possibly misspecified gene trees. *Proceedings of the Royal Society of London. Series B: Biological Sciences*, *269*(1487), 137-142.
- Shimodaira, H. (2002). An approximately unbiased test of phylogenetic tree selection. *Systematic biology*, *51*(3), 492-508.

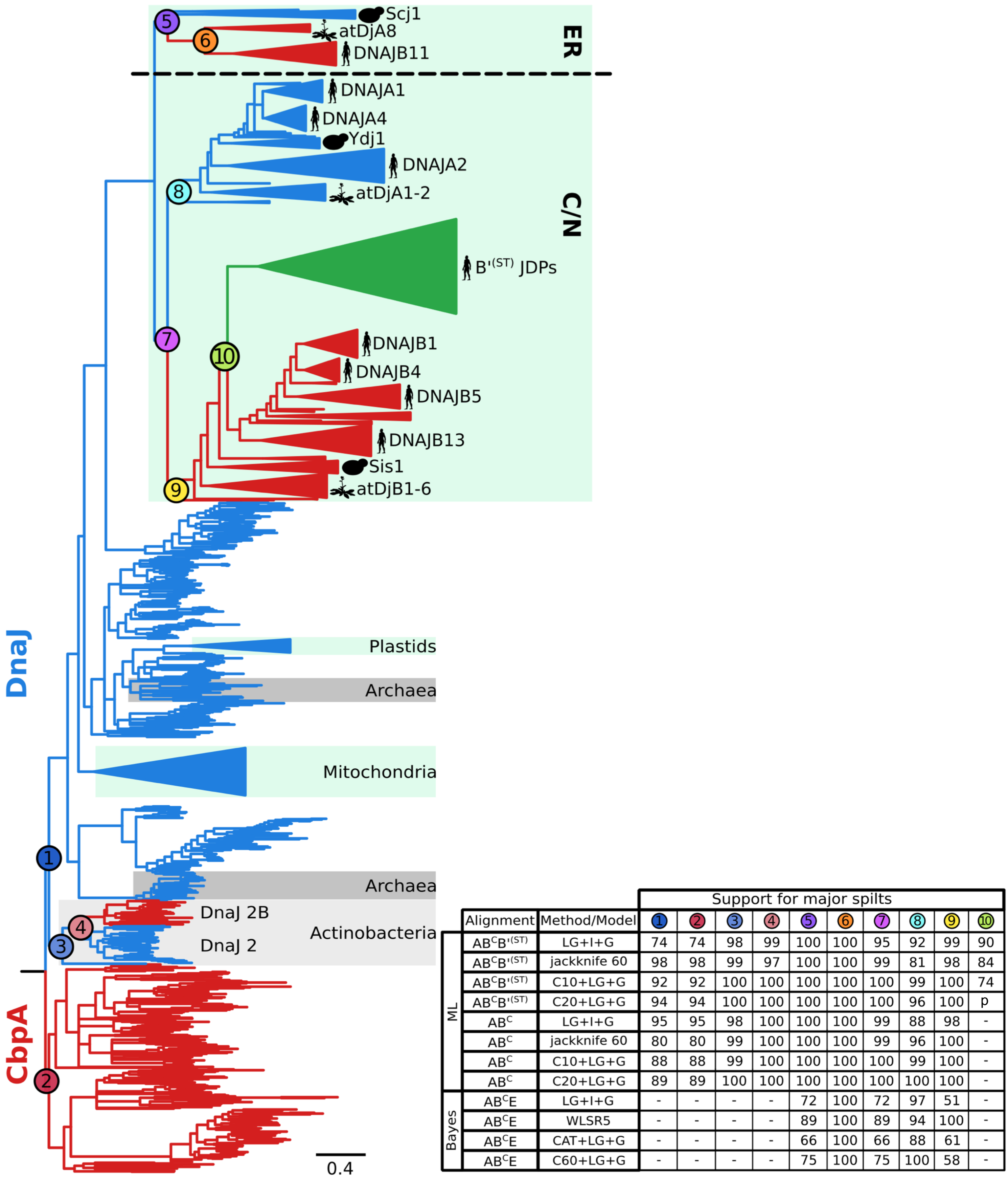


| | Alignment | Method/Model | Support for major spilts | | | | | | | | | |
|---|---|---|---|---|---|---|---|---|---|---|---|---|
| | | | 1 | 2 | 3 | 4 | 5 | 6 | 7 | 8 | 9 | 10 |
| ML | $AB^CB'^{(ST)}$ | LG+I+G | 74 | 74 | 98 | 99 | 100 | 100 | 95 | 92 | 99 | 90 |
| | $AB^CB'^{(ST)}$ | jackknife 60 | 98 | 98 | 99 | 97 | 100 | 100 | 99 | 81 | 98 | 84 |
| | $AB^CB'^{(ST)}$ | C10+LG+G | 92 | 92 | 100 | 100 | 100 | 100 | 100 | 99 | 100 | 74 |
| | $AB^CB'^{(ST)}$ | C20+LG+G | 94 | 94 | 100 | 100 | 100 | 100 | 100 | 96 | 100 | p |
| | $AB^C$ | LG+I+G | 95 | 95 | 98 | 100 | 100 | 100 | 99 | 88 | 98 | - |
| | $AB^C$ | jackknife 60 | 80 | 80 | 99 | 100 | 100 | 100 | 99 | 96 | 100 | - |
| | $AB^C$ | C10+LG+G | 88 | 88 | 99 | 100 | 100 | 100 | 100 | 99 | 100 | - |
| | $AB^C$ | C20+LG+G | 89 | 89 | 100 | 100 | 100 | 100 | 100 | 100 | 100 | - |
| Bayes | $AB^CE$ | LG+I+G | - | - | - | - | 72 | 100 | 72 | 97 | 51 | - |
| | $AB^CE$ | WLSR5 | - | - | - | - | 89 | 100 | 89 | 94 | 100 | - |
| | $AB^CE$ | CAT+LG+G | - | - | - | - | 66 | 100 | 66 | 88 | 61 | - |
| | $AB^CE$ | C60+LG+G | - | - | - | - | 75 | 100 | 75 | 100 | 58 | - |

**Figure S1.** Maximum Likelihood (ML) phylogeny of A, $B^C$ and $B'^{(ST)}$ JDPs ($AB^CB'^{(ST)}$ -alignment) reconstructed using the LG+I+G model. Class A (blue), class $B^C$ (red) class $B'^{(ST)}$ (green). JDPs highlighted: Archaea (dark gray); Actinobacteria (light gray, with additional JDP copies DnaJ2-B and DnaJ2-A indicated); eukaryotic subcellular compartments (light green) - mitochondria, plastids, cytosol/nucleus (C/N), endoplasmic reticulum (ER). The dashed line marks the division between ER and C/N JDPs. Phylogenetic positions of representative class A, $B^C$ $B'^{(ST)}$ JDPs from *Saccharomyces cerevisiae, Arabidopsis thaliana*, and *Homo sapiens* are indicated. Nodes (circles) representing major splits are indicated. Scale bar: amino acid substitutions per position. At right: support for major splits - ML (bootstrap/jackknife 60), Bayes (posterior probability). For tree topologies, see figures S2-S12.

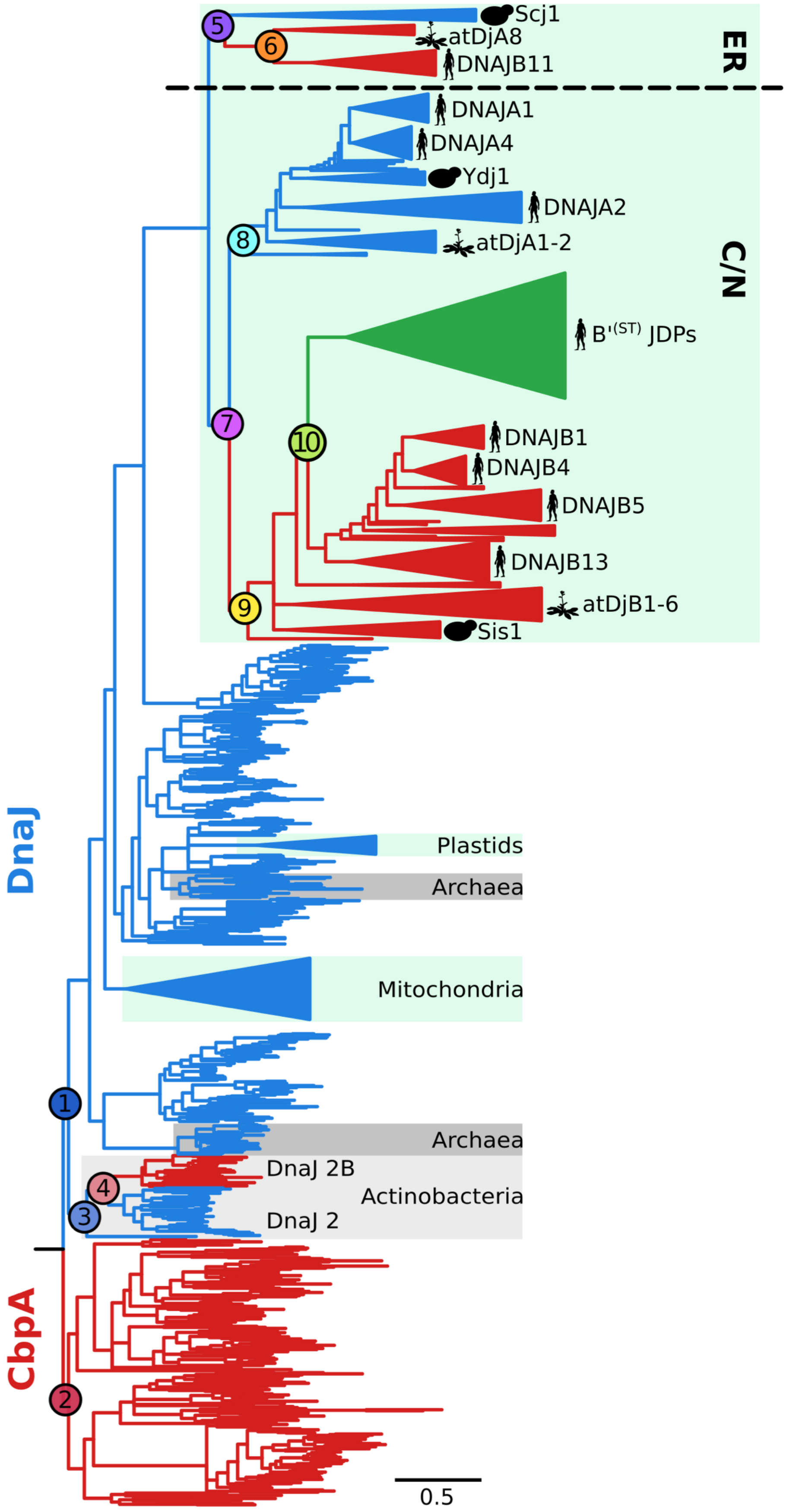


| jackknife 60 | |
|---|---|
| 1 | 98 |
| 2 | 98 |
| 3 | 99 |
| 4 | 97 |
| 5 | 100 |
| 6 | 100 |
| 7 | 99 |
| 8 | 81 |
| 9 | 98 |
| 10 | 84 |

**Figure S2.** Jackknife 60 ML (LG+I+G model) majority-rule consensus tree based on 100 replicates, each comprising 60% of randomly sampled positions from the AB$^{C}$B'$^{(ST)}$-alignment. Color codes and scale bar are as in figure S1. At right: jackknife 60 support for major splits.

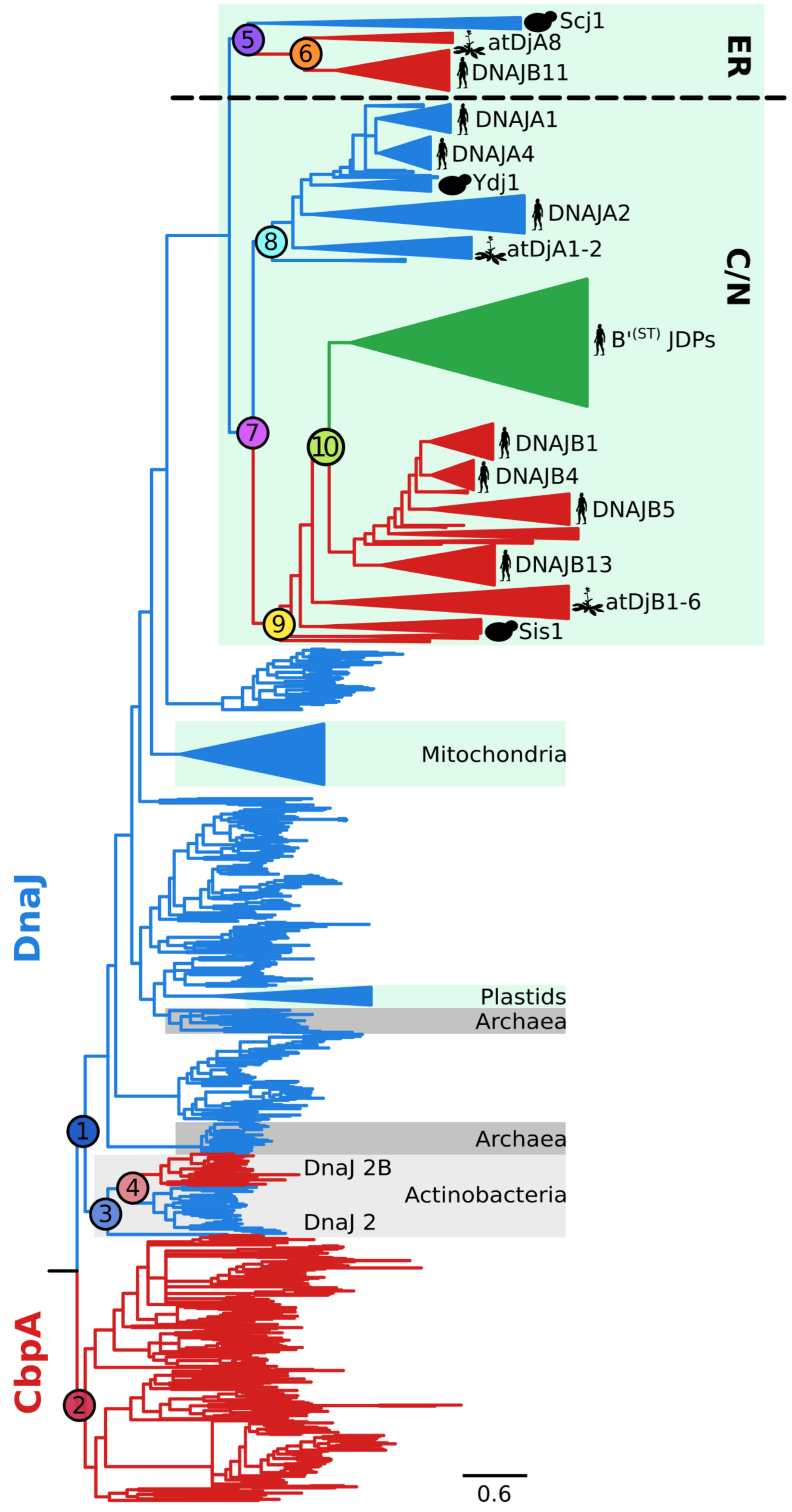


| C10+LG+G | |
|---|---|
| 1 | 92 |
| 2 | 92 |
| 3 | 100 |
| 4 | 100 |
| 5 | 100 |
| 6 | 100 |
| 7 | 100 |
| 8 | 99 |
| 9 | 100 |
| 10 | 74 |

**Figure S3.** ML phylogeny based on $AB^{C}B'^{(ST)}$-alignment, and the C10 + LG +G mixture model of sequence evolution. Color codes and scale bar are as in figure S1. At right: bootstrap support for major splits.

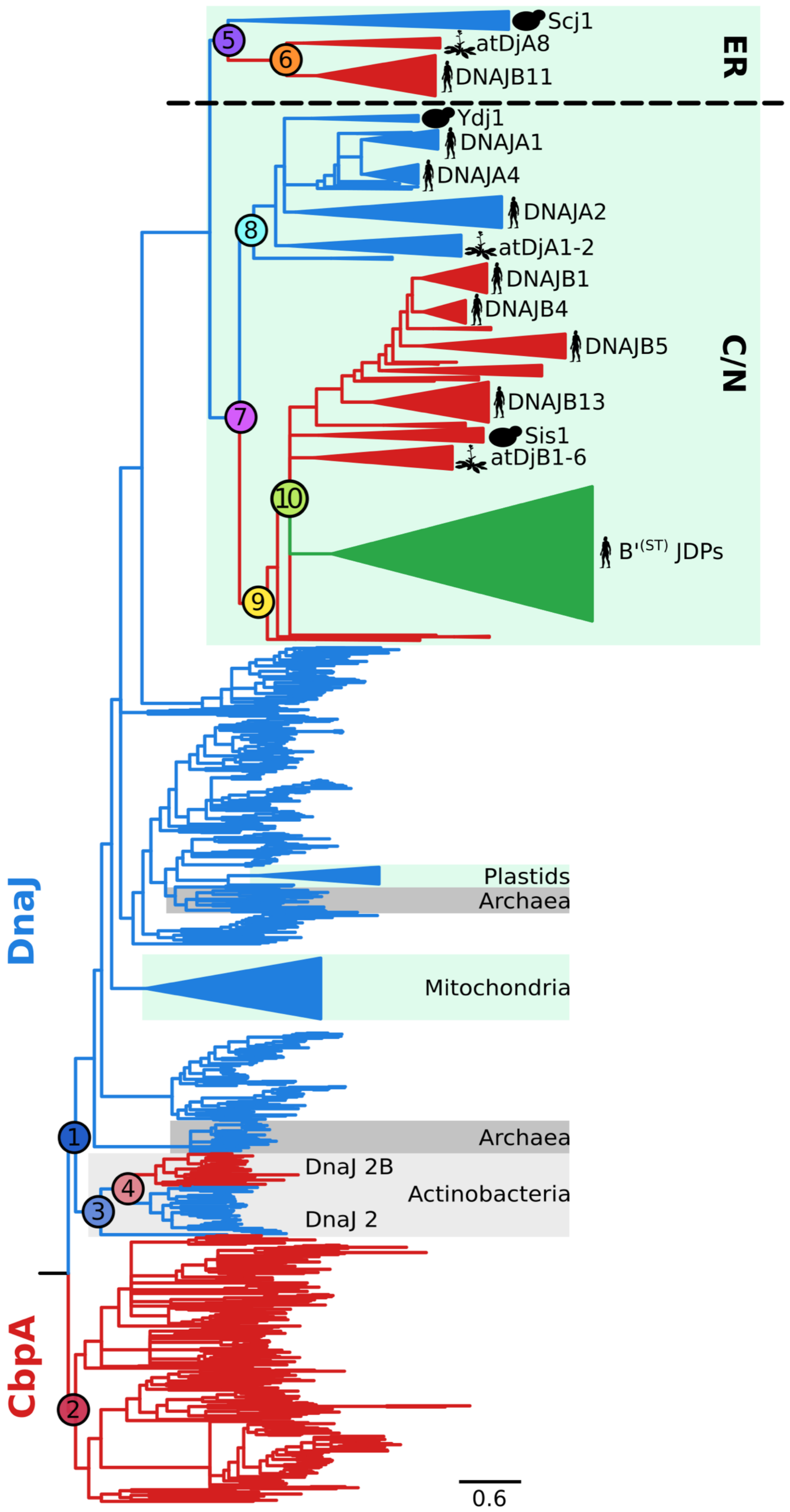


| C20+LG+G | |
|---|---|
| 1 | 94 |
| 2 | 94 |
| 3 | 100 |
| 4 | 100 |
| 5 | 100 |
| 6 | 100 |
| 7 | 100 |
| 8 | 96 |
| 9 | 100 |
| 10 | p |

**Figure S4.** ML phylogeny based on AB$^{C}$B'$^{(ST)}$-alignment and the C20 + LG +G mixture model of sequence evolution. Color codes and scale bar as in figure S1. At right: bootstrap support for major splits. Note polytomy of the B'$^{(ST)}$ split (split 10).

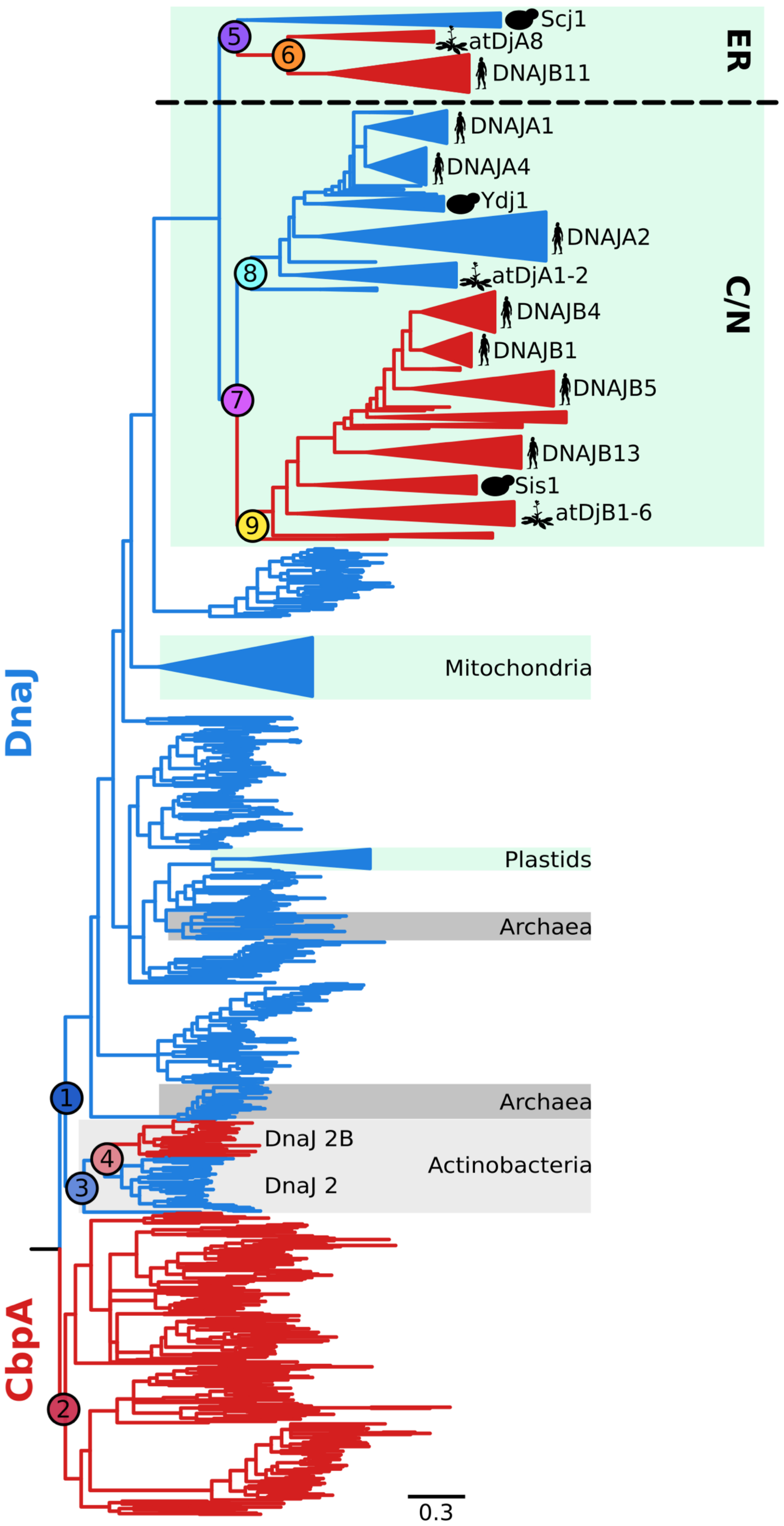


| LG+I+G | |
|---|---|
| 1 | 95 |
| 2 | 95 |
| 3 | 98 |
| 4 | 100 |
| 5 | 100 |
| 6 | 100 |
| 7 | 99 |
| 8 | 88 |
| 9 | 98 |

**Figure S5.** ML phylogeny based on the AB$^{C}$-alignment and the C20+LG+G mixture model of sequence evolution. Color codes and scale bar are as in figure S1. At right: bootstrap support for major splits.

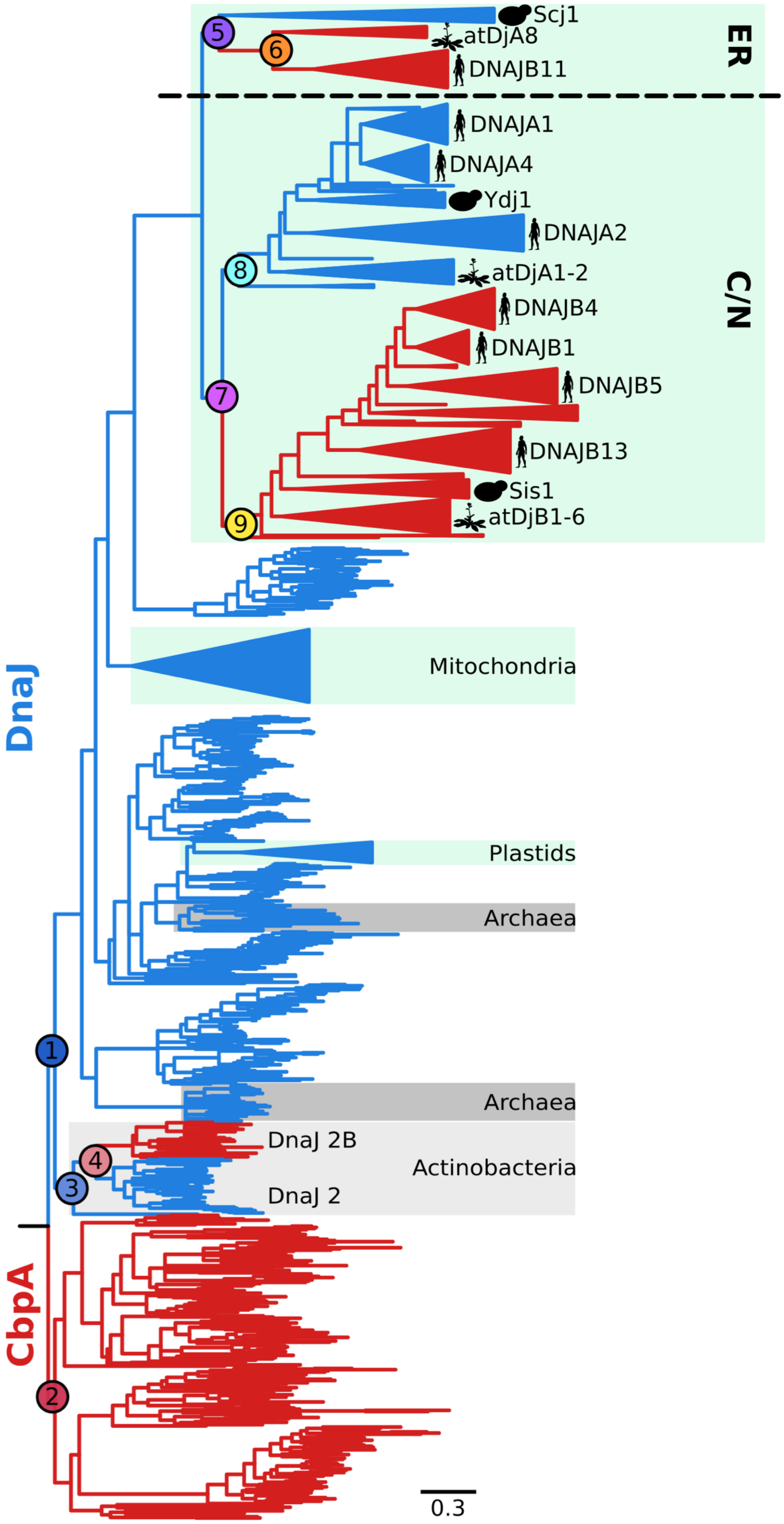


| jackknife 60 | |
|---|---|
| 1 | 80 |
| 2 | 80 |
| 3 | 99 |
| 4 | 100 |
| 5 | 100 |
| 6 | 100 |
| 7 | 99 |
| 8 | 96 |
| 9 | 100 |

**Figure S6.** ML phylogeny based on the AB$^{C}$-alignment and the C10+LG+G mixture model of sequence evolution. Color codes and scale bar are as in figure S1. At right: bootstrap support for major splits.

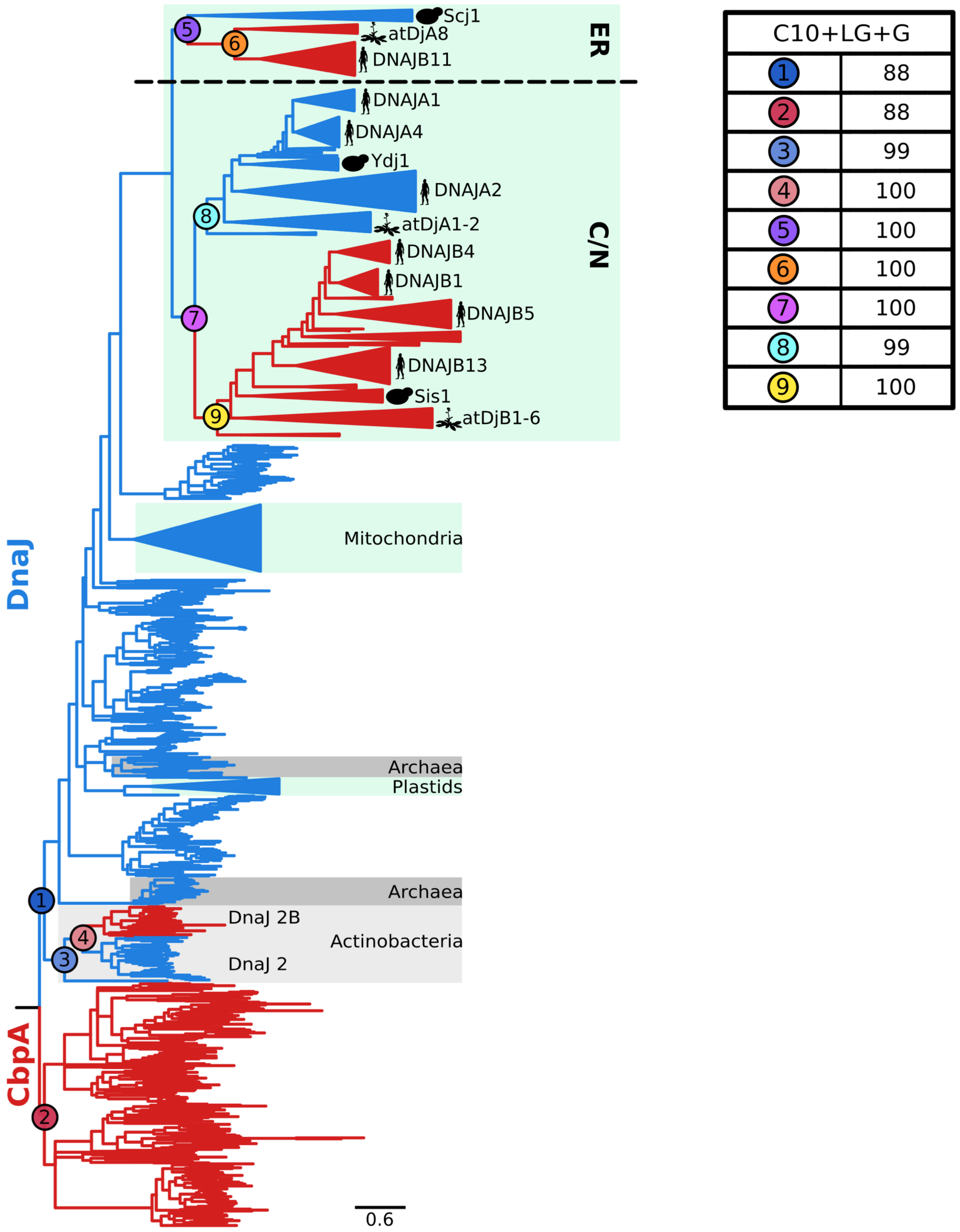


**Figure S7.** Jackknife 60 ML (LG+I+G model) majority-rule consensus tree based on 100 replicates, each comprising 60% of randomly sampled positions from the AB$^{C}$-alignment. Color codes and scale bar are as in figure S1. At right: jackknife 60 support for major splits.

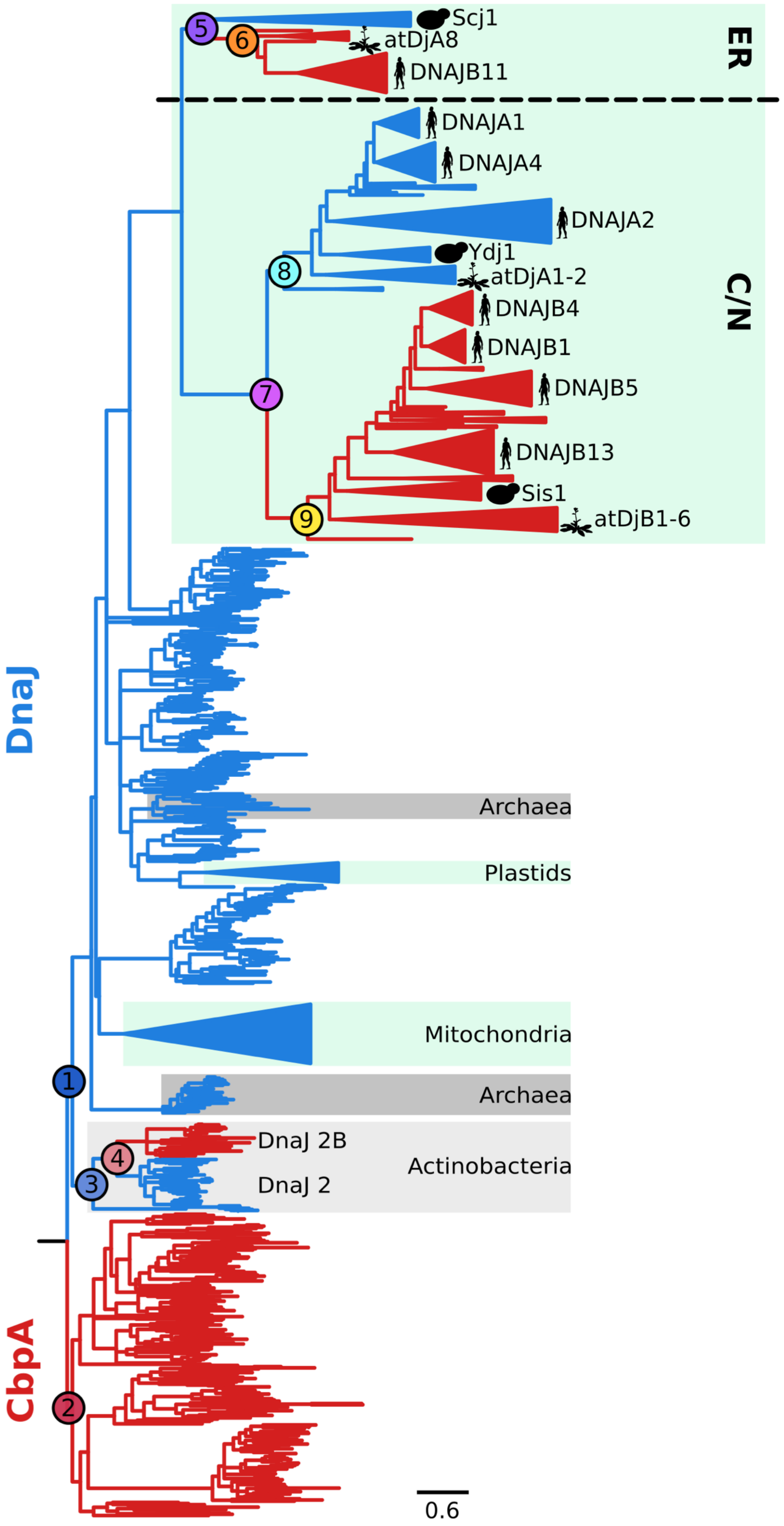


| C20+LG+G | |
|---|---|
| 1 | 89 |
| 2 | 89 |
| 3 | 100 |
| 4 | 100 |
| 5 | 100 |
| 6 | 100 |
| 7 | 100 |
| 8 | 100 |
| 9 | 100 |

**Figure S8.** ML phylogeny based on the AB$^{C}$-alignment and the LG+I+G model of sequence evolution. Color codes and scale bar are as in figure S1. At right: bootstrap support for major splits.

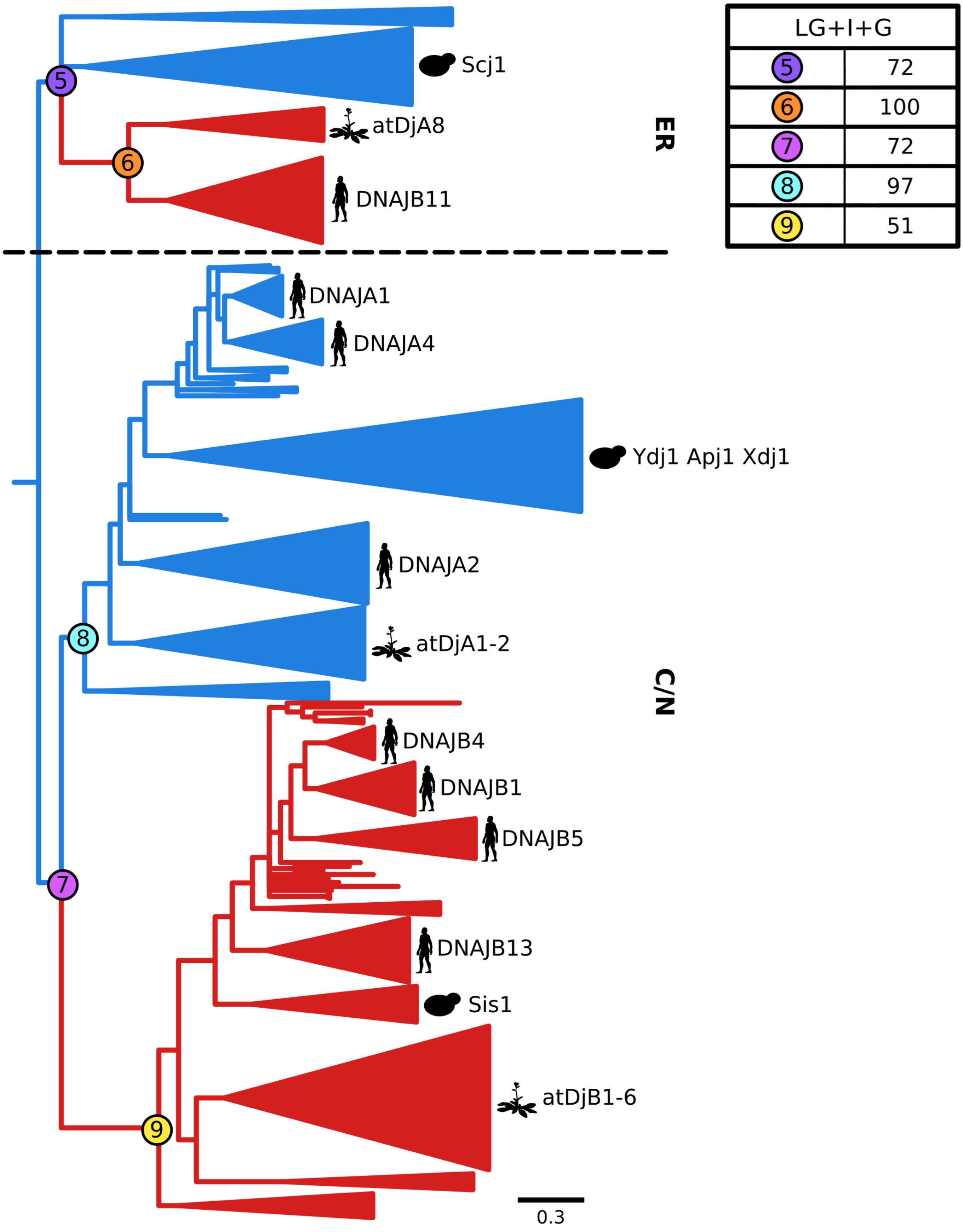


**Figure S9.** Bayesian phylogeny based on the AB$^C$E-alignment and the WLSR5 model of sequence evolution. Color codes and scale bar are as in figure S1. At right: posterior probability for major splits.

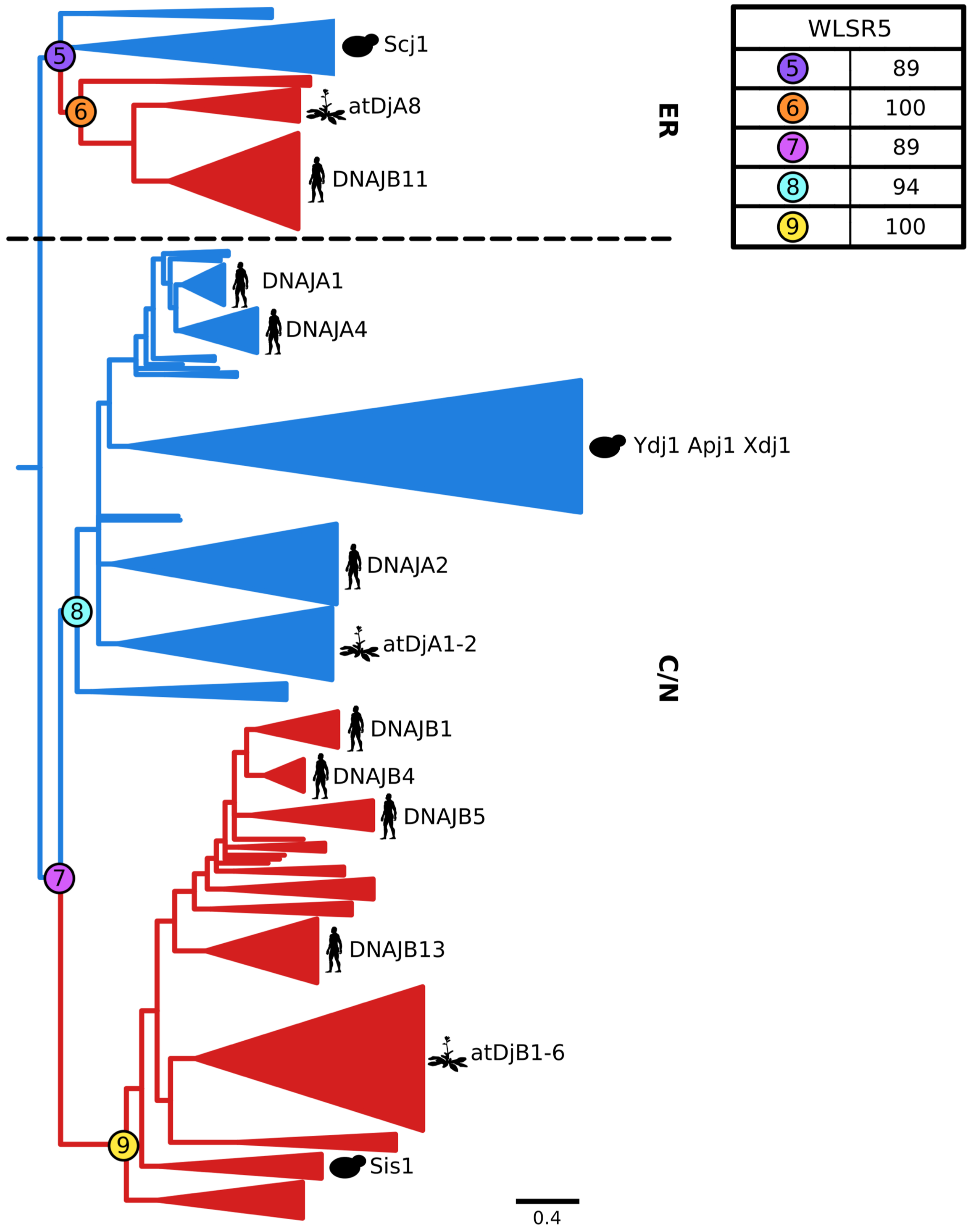


**Figure S10.** Bayesian phylogeny based on the AB$^{C}$E-alignment and the LG+I+G model of sequence evolution. Color codes and scale bar are as in figure S1. At right posterior probability for major splits.

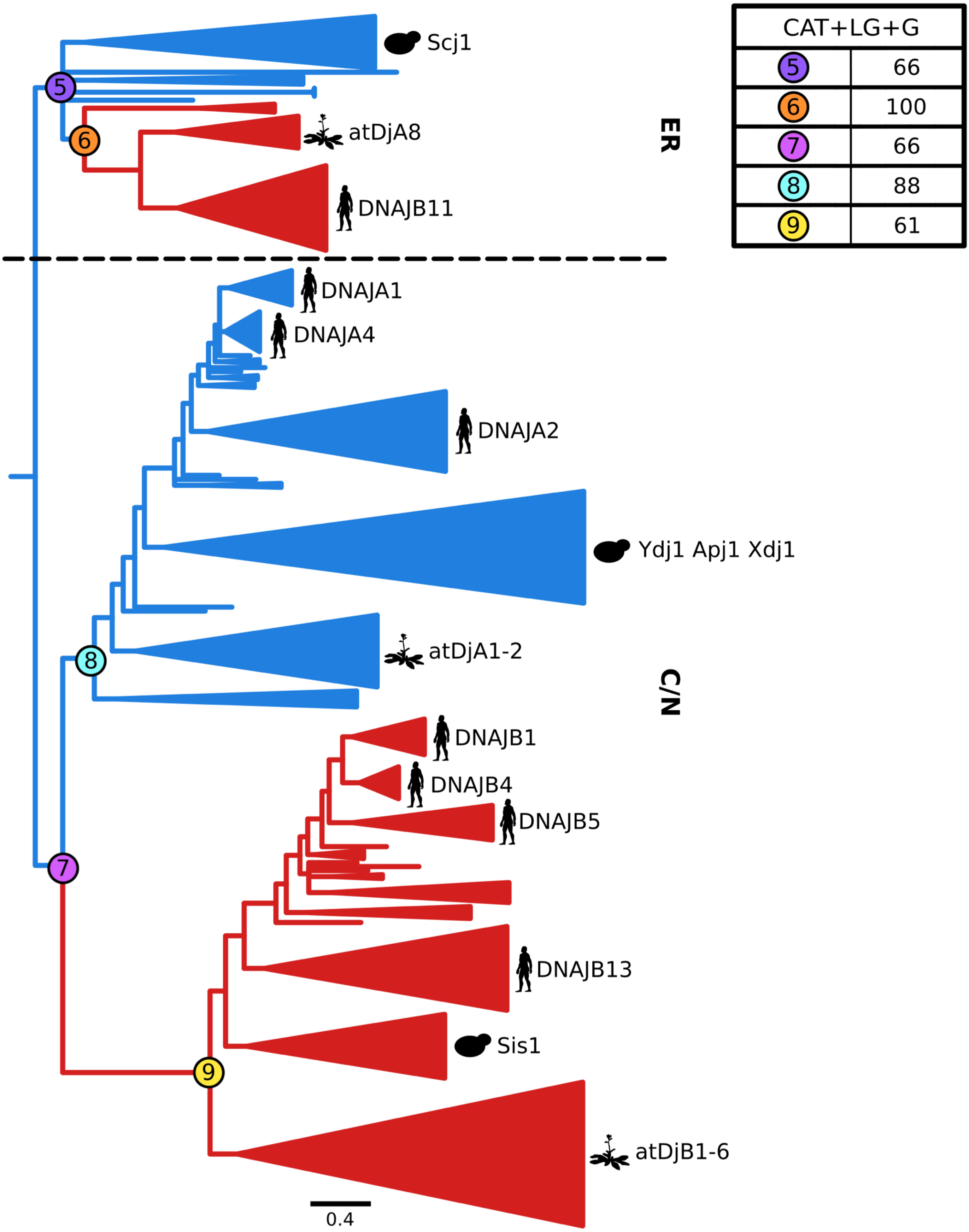


**Figure S11.** Bayesian phylogeny based on the AB$^{C}$E-alignment and the C60+LG+G model of sequence evolution. Color codes and scale bar are as in figure S1. At right: posterior probability for major splits.

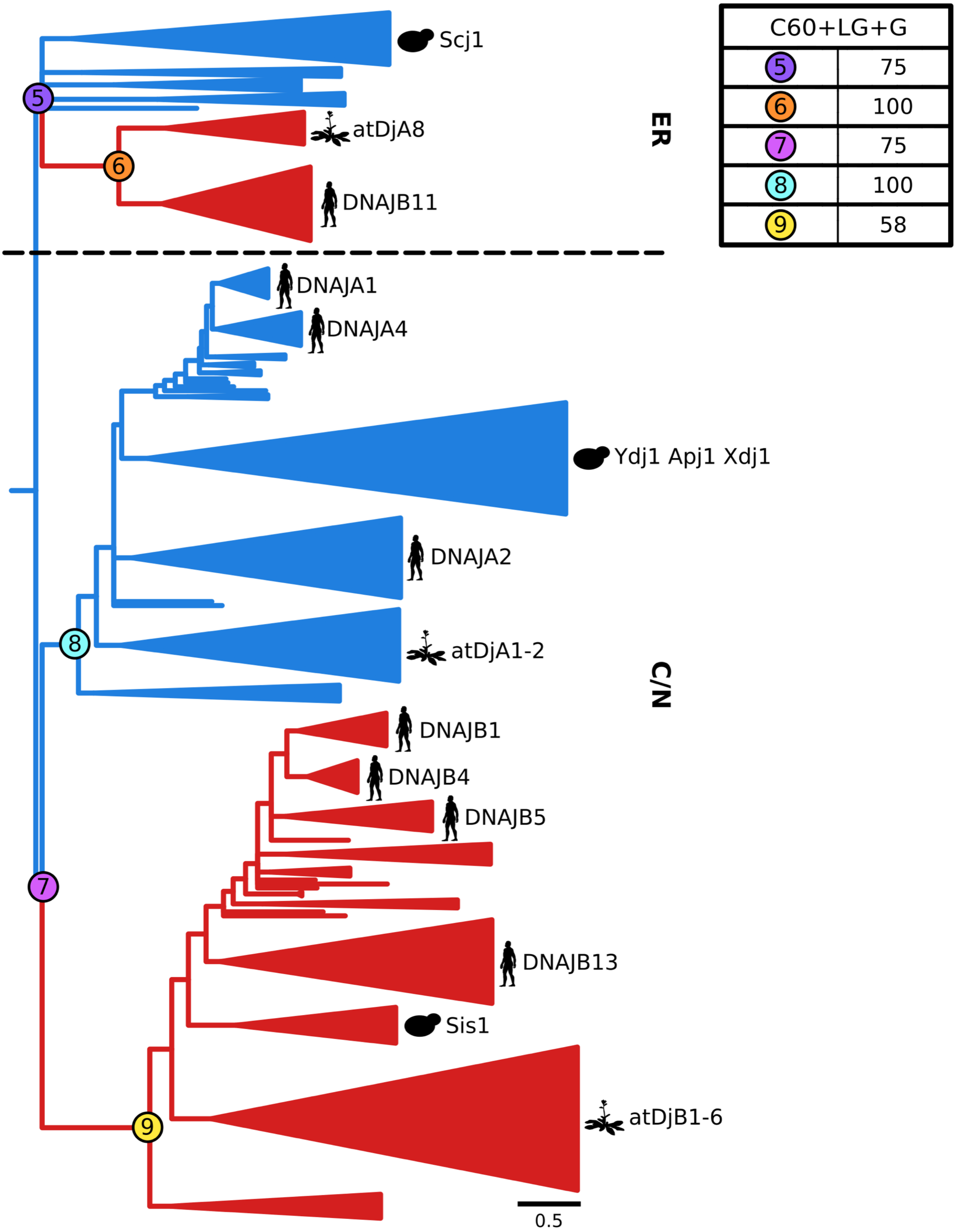


**Figure S12.** Bayesian phylogeny based on the AB$^{C}$E-alignment and the CAT+LG+G model of sequence evolution. Color codes and scale bar are as in figure S1. At right: posterior probability for major splits.

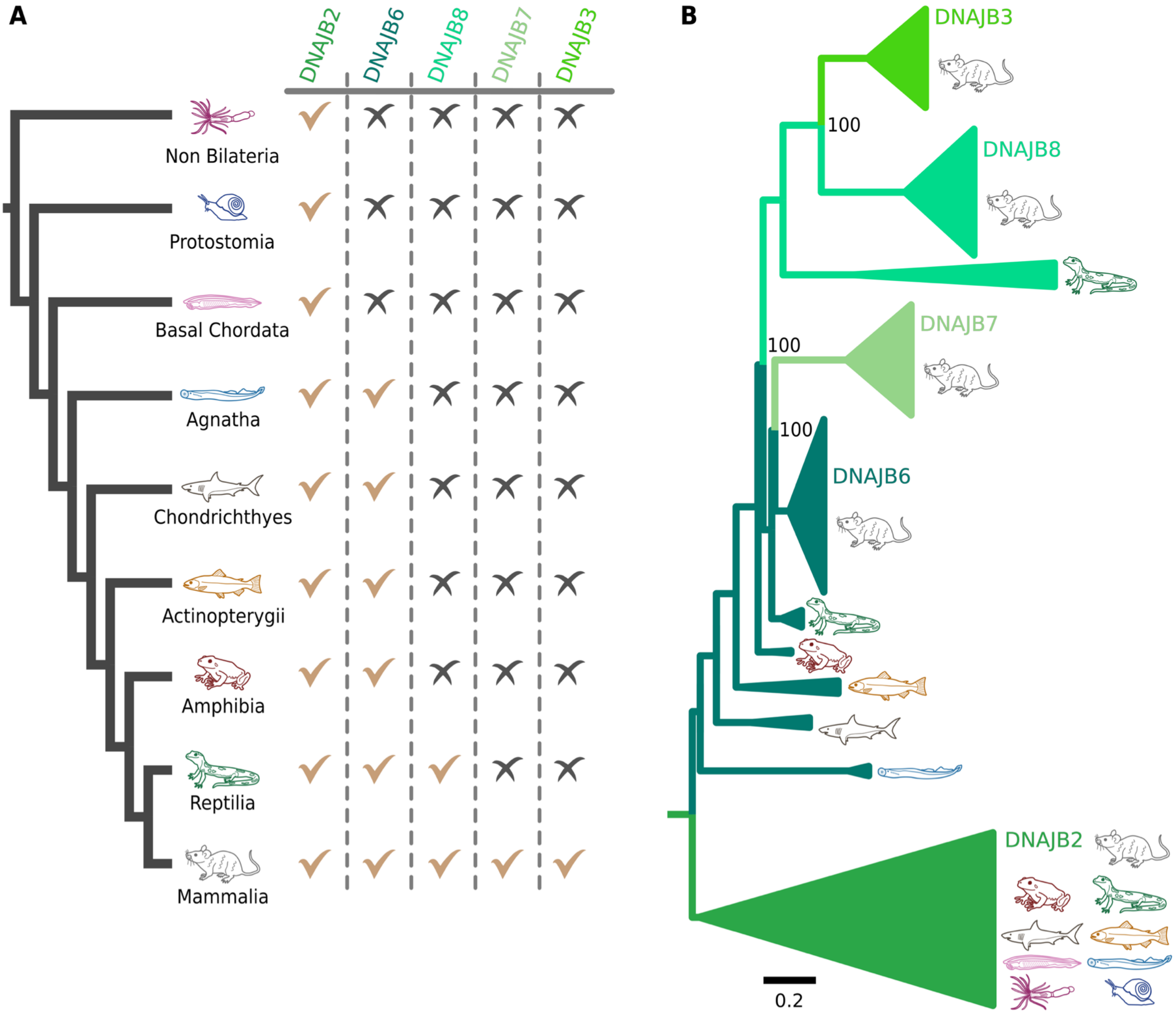


**Figure S13.** Evolution of B'$^{(ST)}$ from the cytosol/nucleus of metazoans. (A) Phylogenetic distribution of B'$^{(ST)}$ JDPs across major metazoan clades; presence (✓) or absence (✗). (B) ML phylogeny based on the B'$^{(ST)}$-alignment and the JTT+I+G model of sequence evolution. The tree was constrained to enforce the monophyly of a clade encompassing DNAJB8/B6/B3 homologs from reptiles and mammals. Each shade of green corresponds to one B'$^{(ST)}$ paralog. Scale bar: amino acid substitutions per position. Bootstrap support for major splits is indicated.

**A**

| | J-domain<br>A | J-domain<br>$B^C$ |
|---|---|---|
| J-domain<br>$B'^{(ST)}$ | $7.6*10^{-45}$<br>214.6 | $1.9*10^{-50}$<br>240.3 |

| | G/F region<br>A | G/F region<br>$B^C$ |
|---|---|---|
| G/F region<br>$B'^{(ST)}$ | $6*10^{-4}$<br>19.6 | $1.3*10^{-10}$<br>47.5 |

**B**

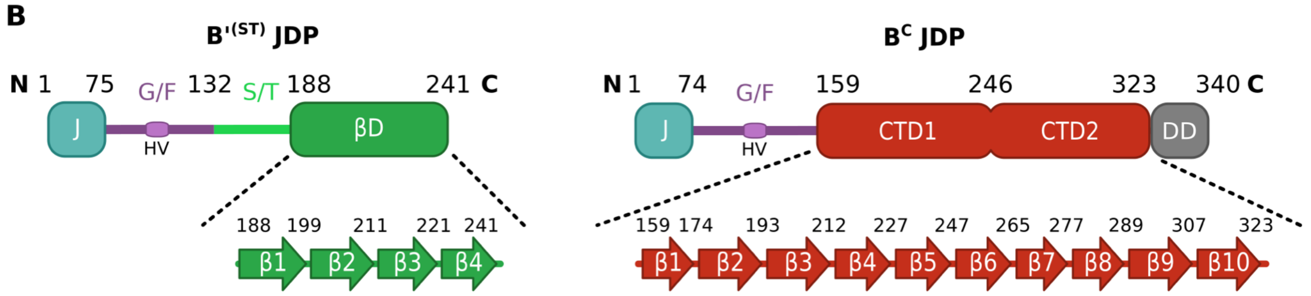


**C**

| | CTD1<br>$B^C$ | CTD2<br>$B^C$ | CTD2\|DD<br>$B^C$ | CTD1\|CTD2<br>$B^C$ | CTD1\|CTD2\|DD<br>$B^C$ |
|---|---|---|---|---|---|
| S/T\|βD<br>$B'^{(ST)}$ | 0.59<br>8.8 | 0.43<br>9.5 | 0.49<br>9.4 | 0.46<br>10.8 | 0.47<br>10.9 |
| S/T<br>$B'^{(ST)}$ | 0.93<br>5.3 | 0.76<br>6.3 | 0.78<br>6.5 | 0.92<br>6.3 | 0.92<br>6.4 |
| βD<br>$B'^{(ST)}$ | 0.3<br>9.3 | 0.11<br>11.2 | 0.12<br>11.3 | 0.25<br>11.0 | 0.26<br>11.1 |

**D**

| | β1<br>$B^C$ | β2<br>$B^C$ | β3<br>$B^C$ | β4<br>$B^C$ | β5<br>$B^C$ | β6<br>$B^C$ | β7<br>$B^C$ | β8<br>$B^C$ | β9<br>$B^C$ | β10<br>$B^C$ |
|---|---|---|---|---|---|---|---|---|---|---|
| β1<br>$B'^{(ST)}$ | 0.31<br>3.0 | 0.18<br>5.1 | 0.35<br>5.1 | 0.20<br>4.3 | 0.55<br>3.0 | 0.55<br>2.8 | 0.20<br>2.6 | 0.073<br>3.1 | 0.25<br>4.2 | 0.41<br>3.5 |
| β2<br>$B'^{(ST)}$ | 0.22<br>3.5 | 0.037<br>7.0 | 0.21<br>5.9 | 0.023<br>6.8 | 0.30<br>3.9 | 0.066<br>5.3 | 0.097<br>3.4 | 0.051<br>3.5 | 0.13<br>5.0 | 0.28<br>4.1 |
| β3<br>$B'^{(ST)}$ | 0.34<br>2.8 | 0.91<br>2.3 | 0.54<br>4.3 | 0.84<br>2.1 | 0.37<br>3.5 | 0.62<br>2.5 | 0.11<br>3.1 | 0.31<br>1.6 | 0.12<br>4.8 | 0.82<br>2.2 |
| β4<br>$B'^{(ST)}$ | 0.32<br>2.6 | 0.13<br>5.0 | 0.23<br>5.2 | 0.64<br>2.5 | 0.32<br>3.3 | 0.55<br>2.4 | 0.039<br>3.7 | 0.023<br>3.7 | 0.20<br>4.0 | 0.098<br>4.7 |

**Figure S14.** Pairwise profile-profile comparisons between $B'^{(ST)}$ and class A and $B^C$ JDPs.
(A) Pairwise profile-profile comparisons of J-domains (left) and G/F regions (right) between $B'^{(ST)}$ and A or $B^C$ JDPs – E-values (top) and HHalign scores (bottom). (B) Line diagrams of $B'^{(ST)}$ and $B^C$ JDPs (structured domains, rectangles; unstructured regions, lines); structural segments analyzed in (C) are indicated. (C) Pairwise profile-profile comparisons of indicated structural segments between $B'^{(ST)}$ and $B^C$ JDPs – E-values (top) and HHalign scores (bottom).

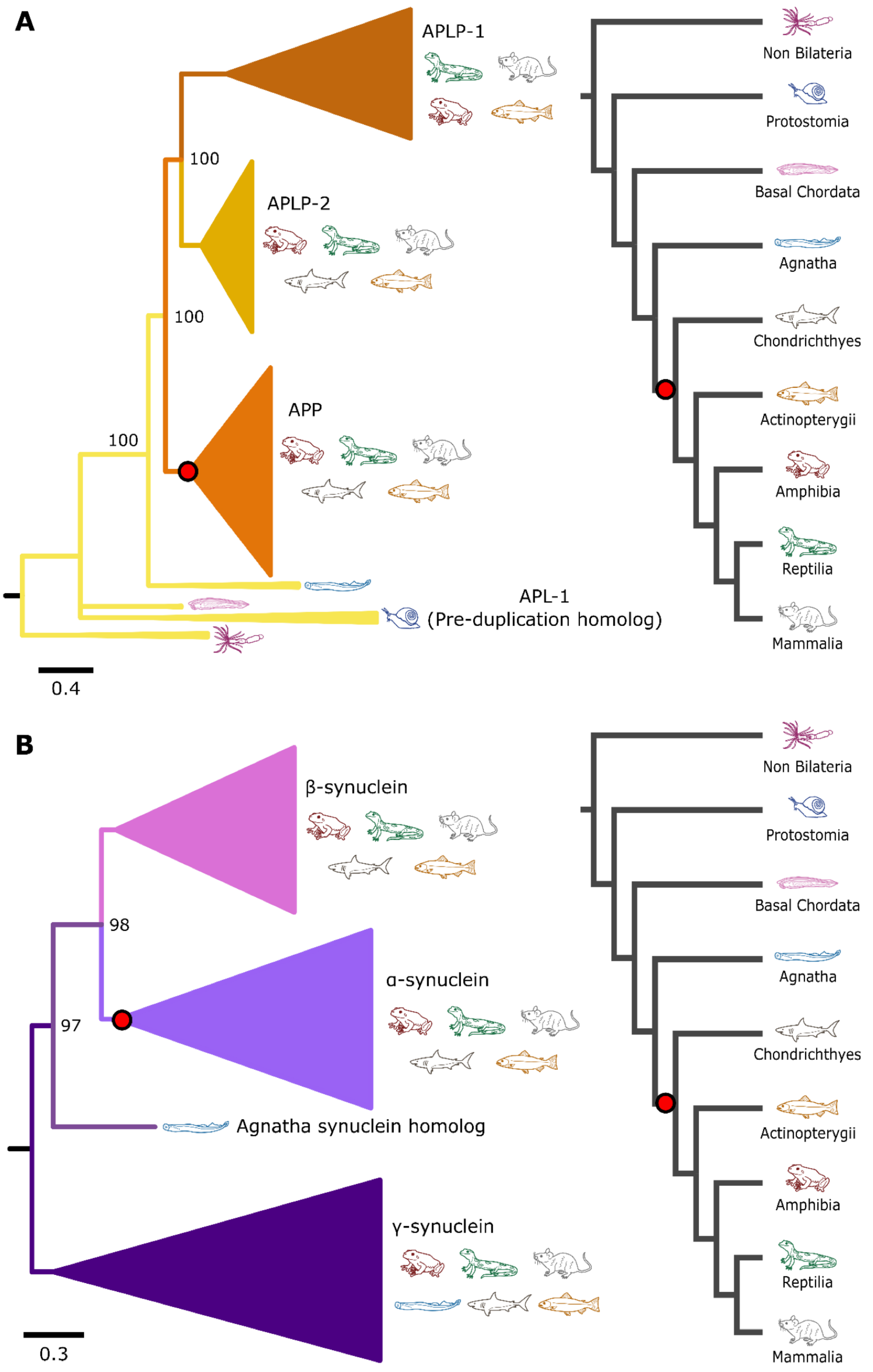


**Figure S15.** Phylogenetic analysis of APP and synuclein homologs from metazoans. Protein trees (left) and simplified metazoan phylogeny (right). The inferred emergence of precursors of human amyloidogenic proteins is indicated by red dots. (A) ML phylogeny of 545 APP homologs: APL-1, APLP-1, APLP-2 and APP, reconstructed using the JTT+I+G model. (B) ML phylogeny of 348 synuclein homologs: α- β- and γ- synuclein and Agnatha synuclein homolog, reconstructed using the JTT+G model. Bootstrap support for major splits is indicated. Splits with support <50 were collapsed into polytomies. Scale bars: amino acid substitutions per position.

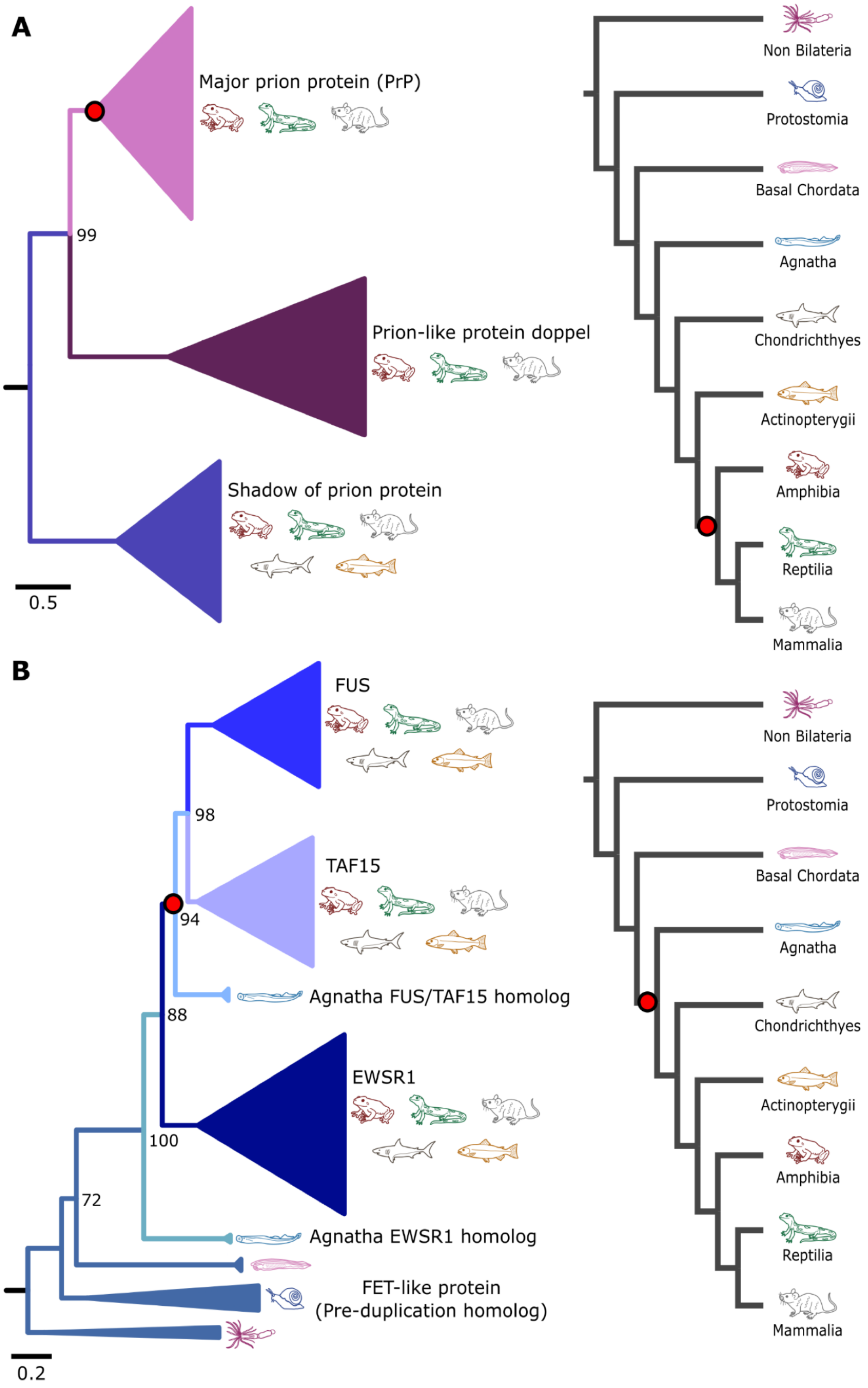


**Figure S16.** Phylogenetic analysis of major prion protein (PrP) and FUS/TAF15 homologs from metazoans. (A) ML phylogeny of 209 major prion protein homologs - PrP, prion like protein doppel, and shadow of prion protein- reconstructed using the JTT+F+G model. (B) ML phylogeny of 426 FUS/TAF15 homologs - FUS, TAF15, EWSR1 and FET like protein - reconstructed using the JTT+F+G model. For other details, see figure S15.

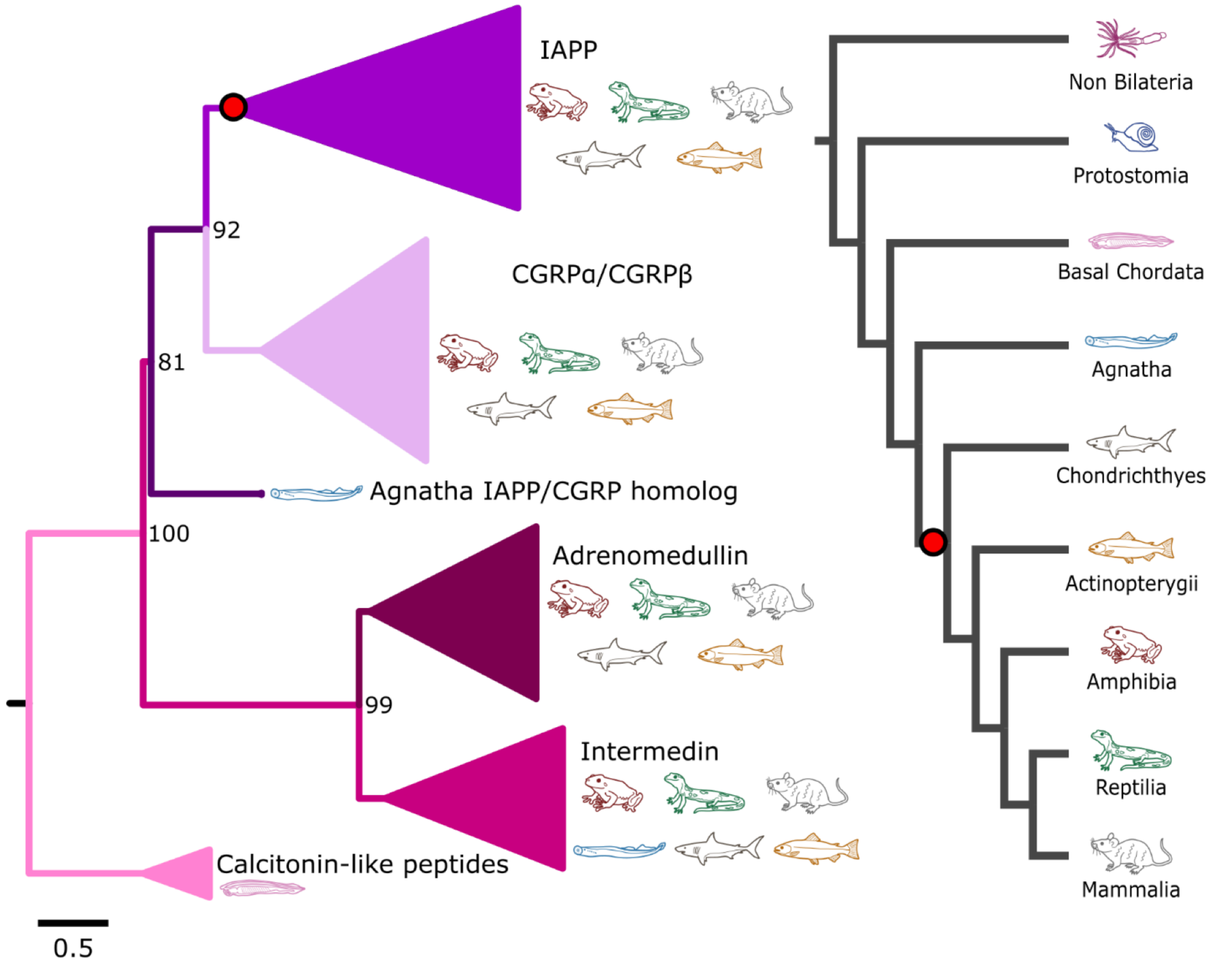


**Figure S17.** Phylogenetic analysis of 514 IAPP homologs: IAPP, CGRPα and β, adrenomedullin, intermedin, and calcitonin like peptides. The ML tree was reconstructed using the JTT+G model. For other details, see figure S15

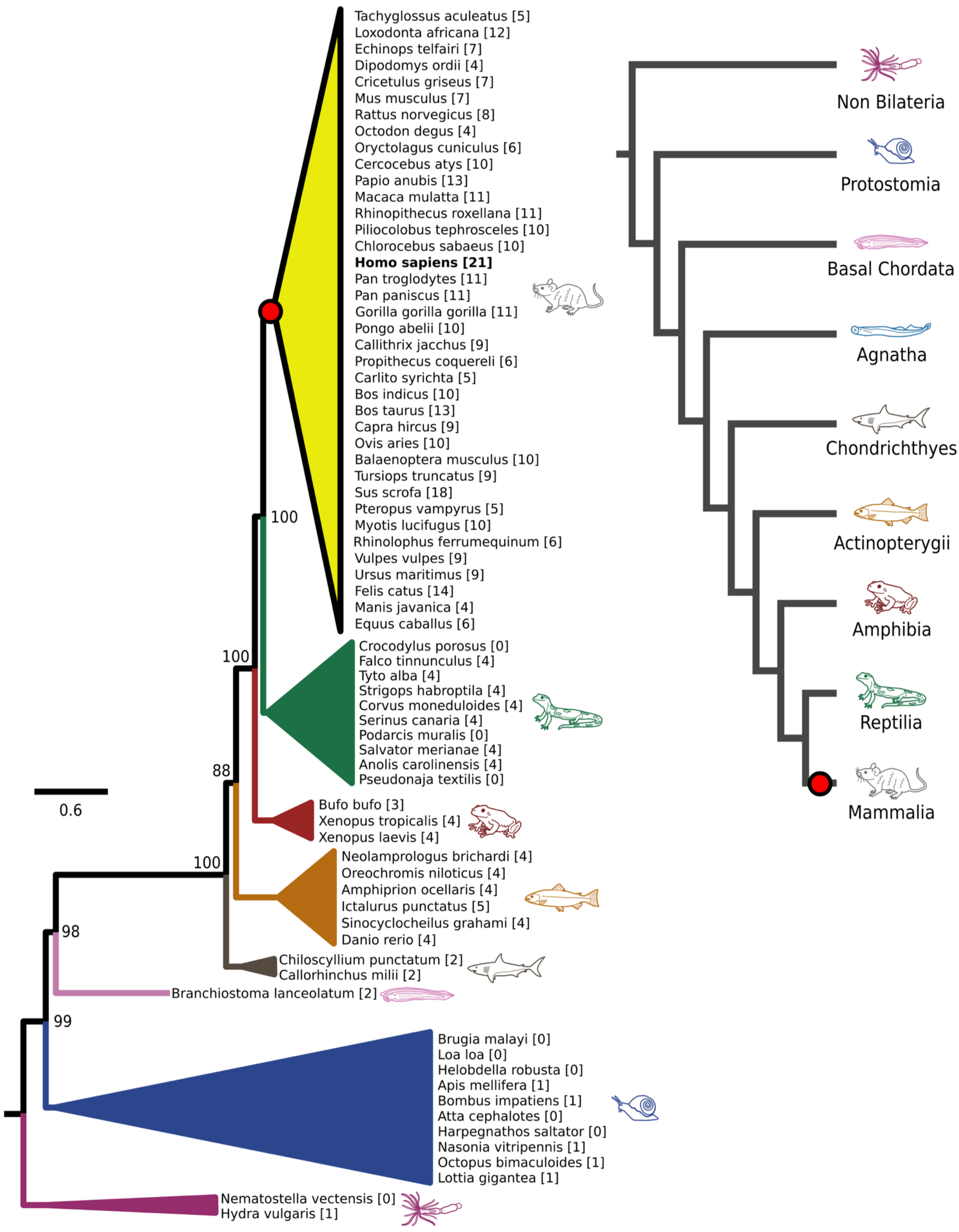


**Figure S18.** Phylogenetic analysis of 59 huntingtin protein homologs from metazoans. The ML tree was reconstructed using the JTT+G model. PolyQ motif lengths are shown in square brackets. For other details, see figure S15.

```
1        JD                                                                       80
DNAJA2   MANVADTKLYDILGVPPGASENELKKAYRKLAKEYHPDKNP---NAGDKFKEISFAYEVLSNPEKRELYDRYGEQGLREG
AncA     --MVKETDYYDILGVSPNASEDEIKKAYRKLALKYHPDKNPDNPEAEEKFKEISEAYEVLSDPEKREIYDQYGEEGLKEG
AncAB    ----MAKDYYDILGVSRGASEDEIKKAYRKLAMKYHPDKNPD-PEAEEKFKEISEAYEVLSDPEKREIYDQYGEEGLKGG
AncB     ----MGKDYYDILGVSRNASEDEIKKAYRKLAMKYHPDKNPD-PEAEEKFKEISEAYEVLSDPEKREIYDQYGEEGLKGG
DNAJB1   ----MGKDYYQTLGLARGASDEEIKRAYRRQALRYHPDKNKE-PGAEEKFKEIAEAYDVLSDPRKREIFDRYGEEGLKGS

81       G/F                                                                     160
DNAJA2   SGGGG--------------------------------------GMDDIFSHIFGGGLFG---------------------
AncA     GGG----GGGGGGGGGFPM--DDFFPGGGGGGG--------GHDAHDIFSQFFGGGGGGGGGS----GFSG---FGGGSD
AncAB    GGG-----GGGGGGGGFPG--DGFFPGGGGGGG--------SHDAHDIFSQFFGGGGGGGGGG----GFSG---FGGGGD
AncB     APPGGGGGGGGPGGSGFPGGFGGFHGGGGGGGGGGGGHGFSSRDPNDIFSQFFGGGGAGGGGFGGSSGFGFGGGGGGGDD
DNAJB1   GPSGGSGGGANGT--SFSYTF--------------------HGDPHAMFAEFFGGRNPF--------------------D

161      G/F                                         CTD1                        240
DNAJA2   ----------------------------------FMGNQSRSRNGRRRGEDMMHPLKVSLEDLYNGKTTKLQLSKNVLCS
AncA     GFFGMRGGGMGGGGGGMGGGG-----------------SRGRPRGPRKGEDIEVPLKVTLEDLYNGKTKKLKITRNVICS
AncAB    GFFGMRGGGMGGGGGGMGGGSGFSSGFRSHSG--GFGAGNGRSRQPRKGPDIEVPLEVTLEDLYNGTTKKLKITRNVICS
AncB     GFFGMRGGGMGGGMGGMGGGGSGFSSGFRSHSGGFGNGSGRSGRQPRKPPPIEVPLPVTLEELYNGTTKKMKITRKRLNA
DNAJB1   TFFGQRNGEEGMDIDDPFSGFPMGMGGFTNVNF-GRSRSAQEPARKKQDPPVTHDLRVSLEEIYSGCTKKMKISHKRLN-

         ZnF                                                             CTD1    320
DNAJA2   ACSGQGGKSG-AVQKCSACRGRGVRIMIRQLAPGMVQQMQSVCSDCNGEGEVINEKDRCKKCEGKKVIKEVKILEVHVDK
AncA     KCNGKGSKGG-AVQTCSTCNGQGMKVVTRQMGPGMVQQMQQTCPKCNGKGEIINEKDRCNACHGKKVVKEKKILEVHIEP
AncAB    KCNGTGAKGG-AVQTCSTCNGQGMKVVTQQMGPGMFQQMQQTCPKCNGKGKII--KDRCKECHGKKVVKEKKILTVNIEP
AncB     S---------------------------------------------------------------GKTVKEDKILTINVKP
DNAJB1   -------------------------------------------------------------PDGKSIRNEDKILTIEVKK

         CTD1                                CTD2                                400
DNAJA2   GMKHGQRITFTGEADQAPGVEPGDIVLLLQEKEHEVFQRDGNDLHMTYKIGLVEALCGFQFTFKHLDGRQIVVKYPPGKV
AncA     GMKDGQKITFPGEGDQ-PGIIPGDIVFVIQEKEHPRFKRKGNDLFMEHTISLTEALCGFTFTIKHLDGRTLTISSDPGDV
AncAB    GMKDGQKITFPGEGDQ-PGGIPGDIVFVIREKPHPRFKRKGNDLFYEVTISLTEALCGFTVTITHLDGRTLKISSDPGDV
AncB     GWKEGTKITFPGEGDQQPGRIPADIVFVIKEKPHPRFKRDGNDLIYTVKISLTEALCGFTVQITTLDGRTLKIPVN--DV
DNAJB1   GWKEGTKITFPKEGDQTSNNIPADIVFVLKDKPHNIFKRDGSDVIYPARISLREALCGCTVNVPTLDGRTIPVVFK--DV

         CTD2                                DD                  CTE             480
DNAJA2   IEPGCV-RVVRGEGMPQYRNPFEKGDLYIKFDVQFPENNWINPDKLSELEDLLPSRPEVPNIIGE-TEE--VE-LQEFDS
AncA     IQPGSV-KCIPGEGMPIHRNPHERGDLYIQFNVEFPNSLT-P-EQLKALKEVLPSSSSPTVPKDDKVEECELE-VDPNQY
AncAB    IQPGSV-KTIPGEGMPIHRNPHQRGDLYIQFNVEFPKSLT-P-EQKKALKEVLPS-------------------------
AncB     IQPGSEKTVIPGEGMPISKNPGQRGDLIIEFNVKFPKSLT-P-EQKKALKEVLPS-------------------------
DNAJB1   IRPGMR-RKVPGEGLPLPKTPEKRGDLIIEFEVIFPERIP--QTSRTVLEQVLPI-------------------------

         CTE                               514
DNAJA2   TRGSGGGQRREAYNDSSDEESSSHHGPGVQCAHQ
AncA     RRSSQQHASREAYDEDDDE--DG--GPGVQCAQQ
AncAB    ----------------------------------
AncB     ----------------------------------
DNAJB1   ----------------------------------
```

**Figure S19.** Sequence alignment of reconstructed ancestral JDPs. J - J-domain, teal; G/F - glycine/phenylalanine rich region, violet; CTD1/CTD2 - substrate binding domains, blue; ZnF - zinc finger, yellow; DD - dimerization domain, light gray, CTE - C-terminal extension, dark gray.

**A**

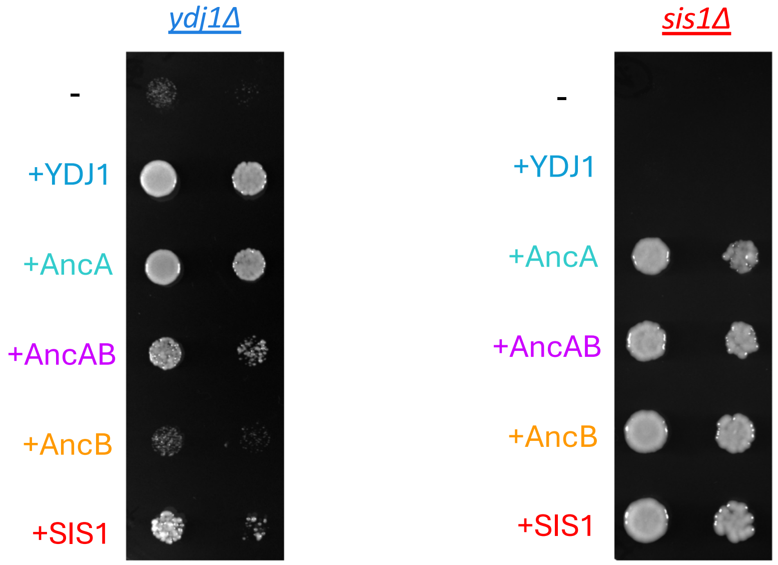


**B**

Statistical analysis Fig. 4C

| | No addition | - JDP | DNAJA2 | AncA | AncAB | AncB | DNAJB1 |
|---|---|---|---|---|---|---|---|
| No addition | - | ns | **** | ** | *** | **** | **** |
| - JDP | ns | - | **** | ** | ** | **** | *** |
| DNAJA2 | **** | **** | - | * | ns | ns | ns |
| AncA | ** | ** | * | - | ns | * | ns |
| AncAB | *** | ** | ns | ns | - | * | ns |
| AncB | **** | **** | ns | * | * | - | ns |
| DNAJB1 | **** | *** | ns | ns | ns | ns | - |

**Figure S20.** Initial assessment of activity of ancestral JDPs (Fig. 4 B, C). (A) Selection for strains lacking WT *SIS1* or *YDJ1*: *ydj1*Δ (left) and *sis1*Δ (right): 5000 cells of transformants (and a 10 fold dilution, right) harboring plasmid-borne copies of an ancestral JDP, WT *YDJ1*, WT *SIS1* or empty vector (-) were spotted onto solid synthetic medium containing 5-FOA and incubated for 3 days at 30 °C. (B) Results of Tukey's multiple comparison test for luciferase refolding activities in Fig. 4C: p < 0.05 (*), p < 0.01 (**), p < 0.001 (***), p < 0.0001 (****), ns, not significant.

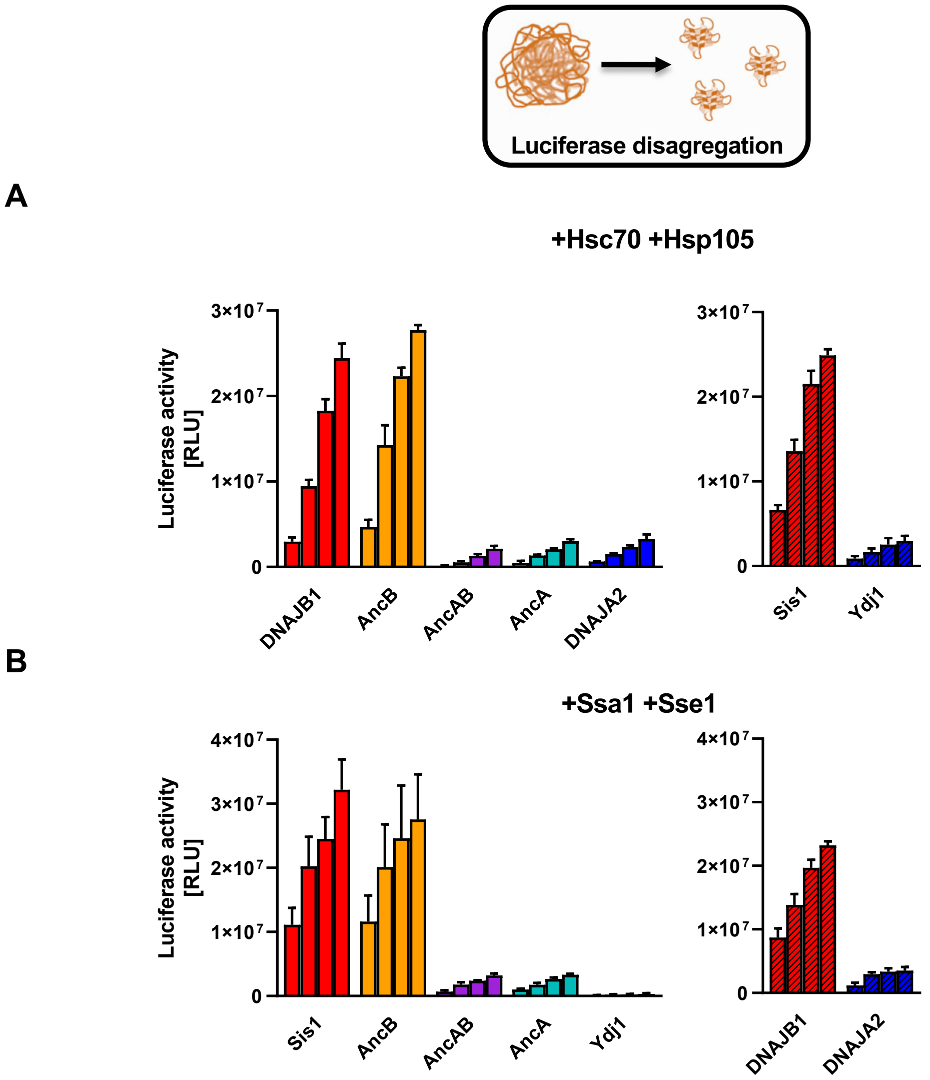


**Figure S21.** Disaggregation of 200 nM luciferase aggregates in the presence of human and/or yeast chaperones. (A) Hsc70 (1.5 μM), Hsp105 (0.15 μM) and JDP (1 μM). (B) Ssa1 (1.5 μM), Sse1 (0.1 μM) and JDP (1 μM). Activity was measured after 1, 2, 3 and 4 hours; average activity of 3 repeats ± standard deviation is shown.

**A**

Statistical analysis Fig. 4D, S21A

| | DNAJA2 | AncA | AncAB | AncB | DNAJB1 |
|---|---|---|---|---|---|
| DNAJA2 | - | ns | ns | **** | **** |
| AncA | ns | - | ns | **** | **** |
| AncAB | ns | ns | - | **** | **** |
| AncB | **** | **** | **** | - | ** |
| DNAJB1 | **** | **** | **** | ** | - |

**B**

Statistical analysis Fig. 4E

| | - JDP | DNAJA2 | AncA | AncAB | AncB | DNAJB1 |
|---|---|---|---|---|---|---|
| - JDP | - | ns | ns | * | **** | **** |
| DNAJA2 | ns | - | ns | * | **** | **** |
| AncA | ns | ns | - | ns | *** | *** |
| AncAB | * | * | ns | - | ** | ** |
| AncB | **** | **** | **** | ** | - | ns |
| DNAJB1 | **** | **** | **** | ** | ns | - |

**C**

Statistical analysis Fig. 4F

| | - JDP | DNAJA2 | AncA | AncAB | AncB | DNAJB1 |
|---|---|---|---|---|---|---|
| - JDP | - | * | ns | ns | **** | **** |
| DNAJA2 | * | - | ns | ns | **** | ** |
| AncA | ns | ns | - | ns | **** | *** |
| AncAB | ns | ns | ns | - | **** | *** |
| AncB | **** | **** | **** | **** | - | ns |
| DNAJB1 | **** | ** | *** | *** | ns | - |

**Figure S22.** Results of Tukey's multiple comparison test for (A) disaggregation of luciferase aggregates in Fig. 4D; (B) disassembly of α-synuclein fibrils in Fig. 4E; (C) recruitment of Hsp70/NEF to Aβ42 amyloid fibrils in Fig. 4F. (B, C) statistical analyses were carried out at the end point of α-synuclein fibril dissociation (700 minutes) and Aβ42 association (30 minutes): $p < 0.05$ (*), $p < 0.01$ (**), $p < 0.001$ (***), $p < 0.0001$ (****), ns, not significant.

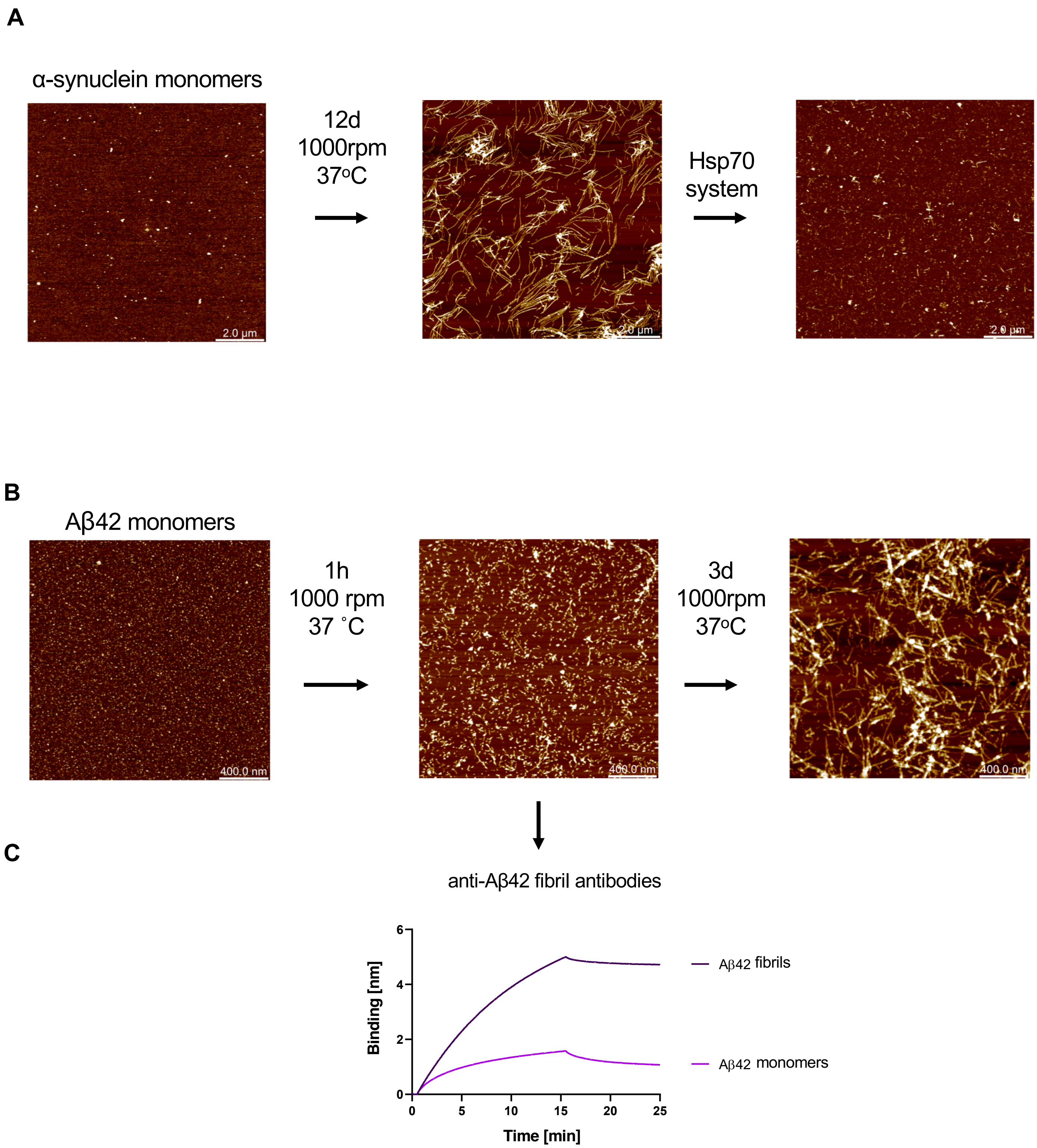


**Figure S23.** Preparation of amyloid fibrils for biochemical experiments. (A) AFM images of α-synuclein recorded (left) prior to fibril formation: (center) after fibril formation (12 days at 37°C with shaking); (right) after disaggregation with Hsc70 (3 µM), DNAJB1 (0.25 µM) and Hsp105 (0.3 µM). (B) Aβ42 fibrils for BLI biosensor immobilization. AFM images taken at three steps of Aβ42 fibrils formation: (left) mixture of WT and biotinylated Aβ42 peptides (at 10:1 molar ratio) prior to fibril formation; (middle) fibrils formed during 1 hour incubation with shaking at 37 °C – these fibrils were immobilized to the BLI sensor; (right) fibrils formed during 3 days of incubation with shaking at 37 °C. (C) Aβ42 fibrils immobilized to the BLI sensor, but not monomeric Aβ42 peptide, bind anti-Aβ42 fibril antibodies.

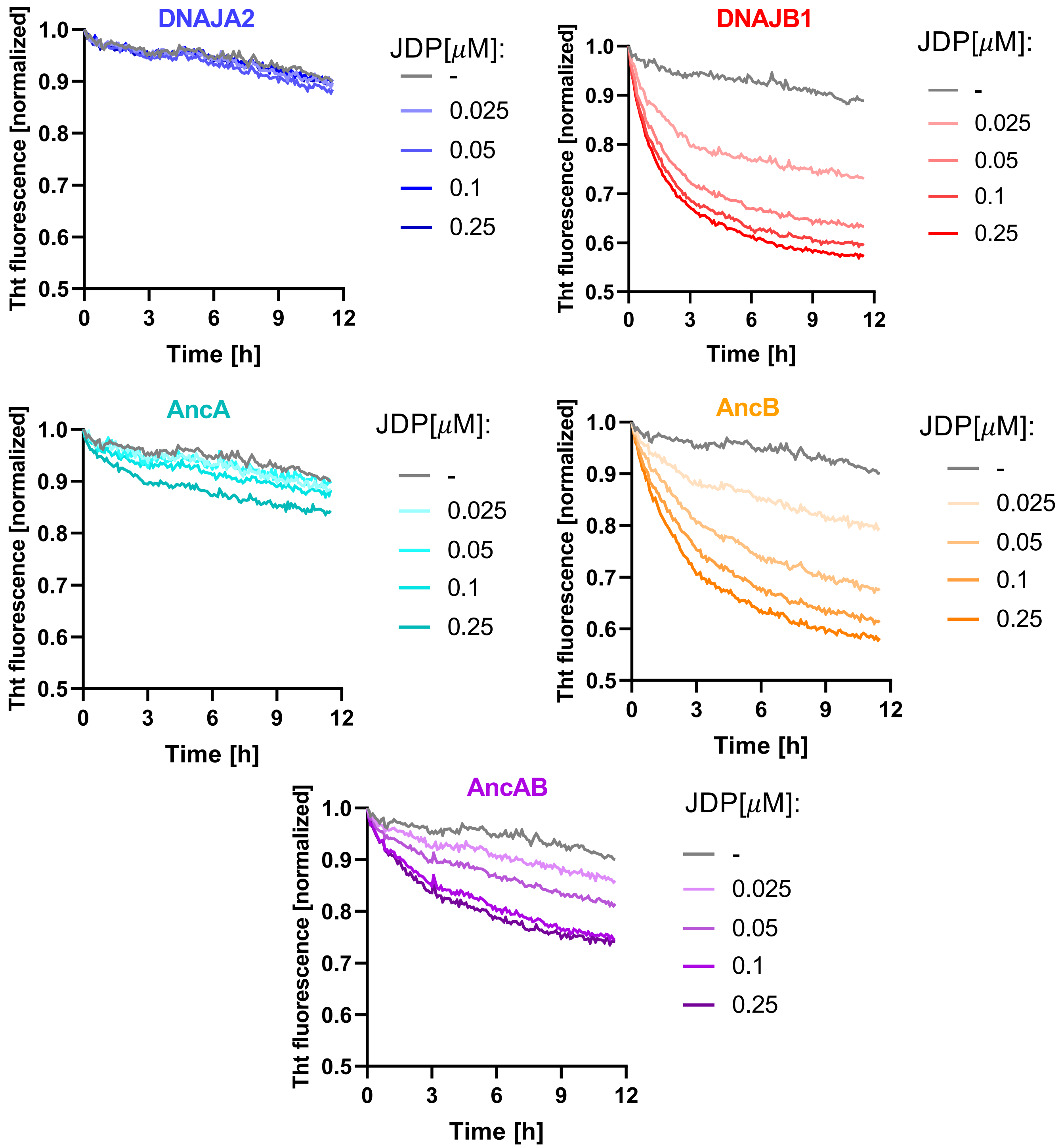


**Figure S24.** Disaggregation of α-synuclein fibrils by ancestral and contemporary JDPs.
Disassembly of α-synuclein fibrils monitored by ThT fluorescence. Reaction mixtures contained α-synuclein fibrils (0.8 μM), Hsc70 (3 μM), Hsp105 (0.3 μM) and the indicated concentrations of JDPs. Data from the control experiment (no JDP added) are the same in each graph. The traces represent the mean values from three or four independent experiments.

**A**

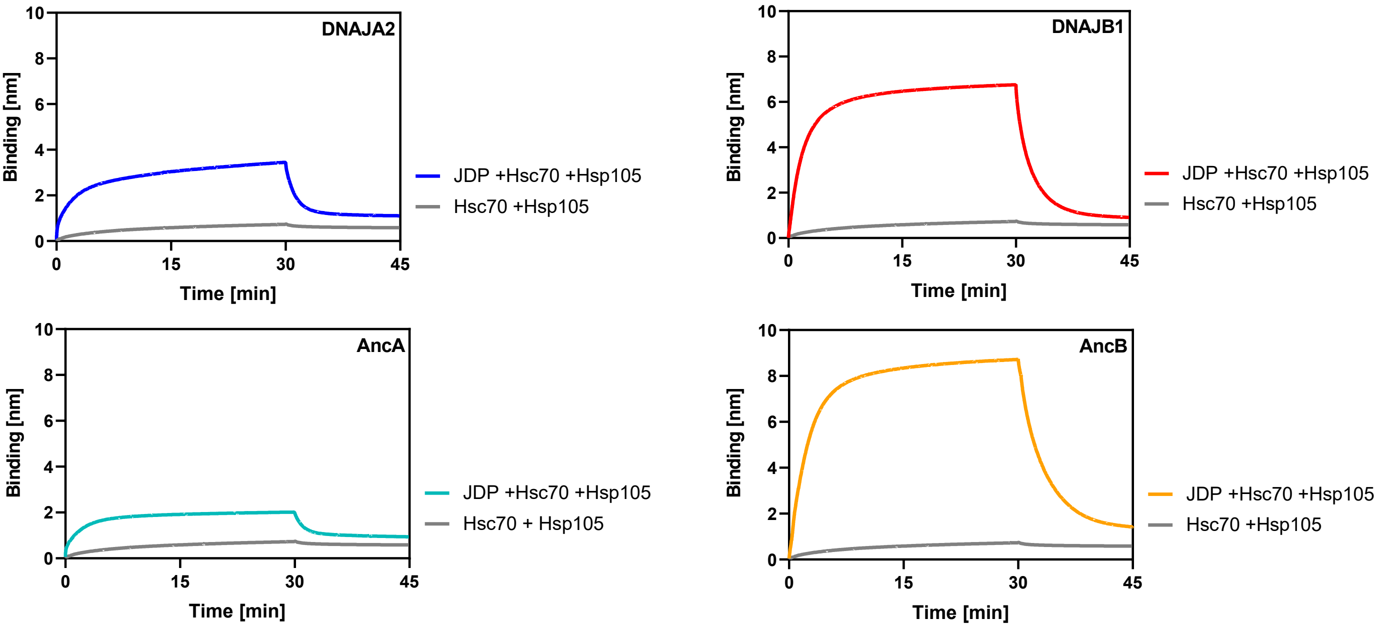


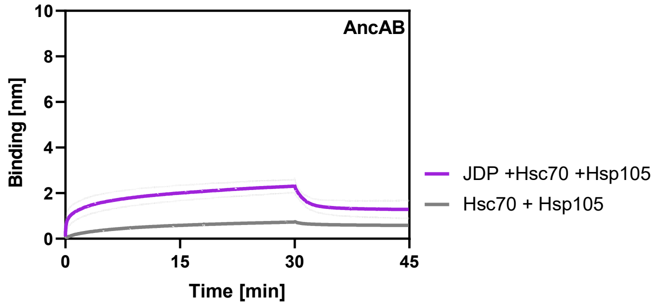


| | - JDP | DNAJA2 | AncA | AncAB | AncB | DNAJB1 |
|---|---|---|---|---|---|---|
| - JDP | - | * | ns | ns | **** | **** |
| DNAJA2 | * | - | ns | ns | **** | ** |
| AncA | ns | ns | - | ns | **** | *** |
| AncAB | ns | ns | ns | - | **** | *** |
| AncB | **** | **** | **** | **** | - | ns |
| DNAJB1 | **** | ** | *** | *** | ns | - |

**B**

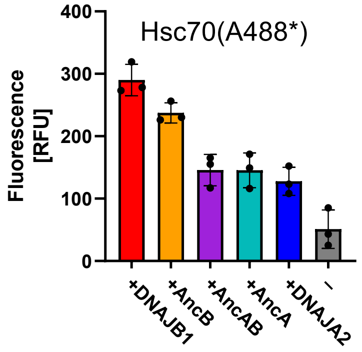


| | - JDP | DNAJA2 | AncA | AncAB | AncB | DNAJB1 |
|---|---|---|---|---|---|---|
| - JDP | - | * | ** | ** | **** | **** |
| DNAJA2 | * | - | ns | ns | ** | **** |
| AncA | ** | ns | - | ns | ** | *** |
| AncAB | ** | ns | ns | - | ** | *** |
| AncB | **** | ** | ** | ** | - | ns |
| DNAJB1 | **** | **** | *** | *** | ns | - |

**Figure S25.** Ancestral JDP-driven recruitment of Hsc70/Hsp105 to Aβ42 amyloid fibrils. (A) Interaction of Hsc70 (1 µM) and Hsp105 (0.1 µM) with BLI biosensor immobilized Aβ42 amyloid fibrils was monitored in the absence (gray) or presence (colored) of indicated ancestral and contemporary JDPs (1 µM). (bottom – right) Tukey's multiple comparison test was carried out at the end time point (30 minutes) of the association phase. (B) (left) Levels of Hsc70 interacting with Aβ42 amyloid fibrils were quantified using fluorescently labeled Hsc70 (A488*-Hsc70). (right) Tukey's multiple comparison test: $p < 0.05$ (*), $p < 0.01$ (**), $p < 0.001$ (***), $p < 0.0001$ (****), ns, not significant

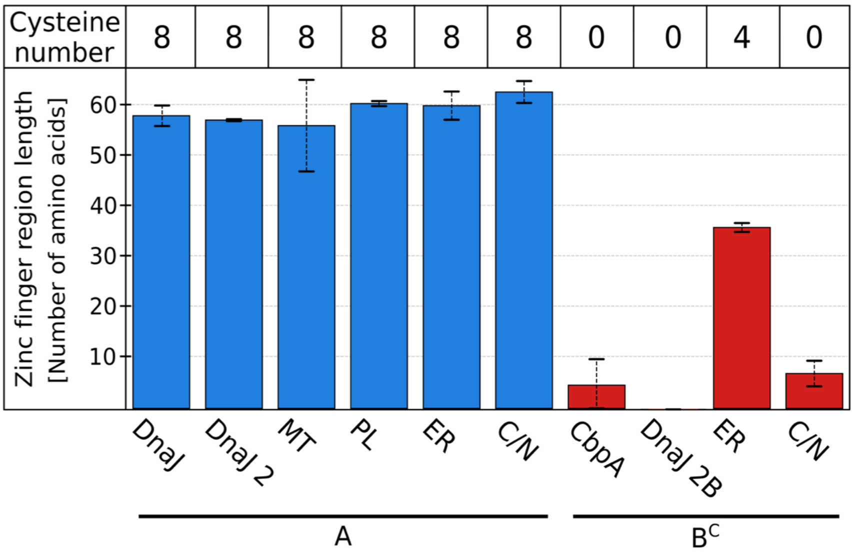


**Figure S26**. Distribution of ZnF length and cysteine content across class A (blue) and $B^C$ (red) JDPs in our dataset. The number of cysteine residues within the ZnF is indicated above the bars.

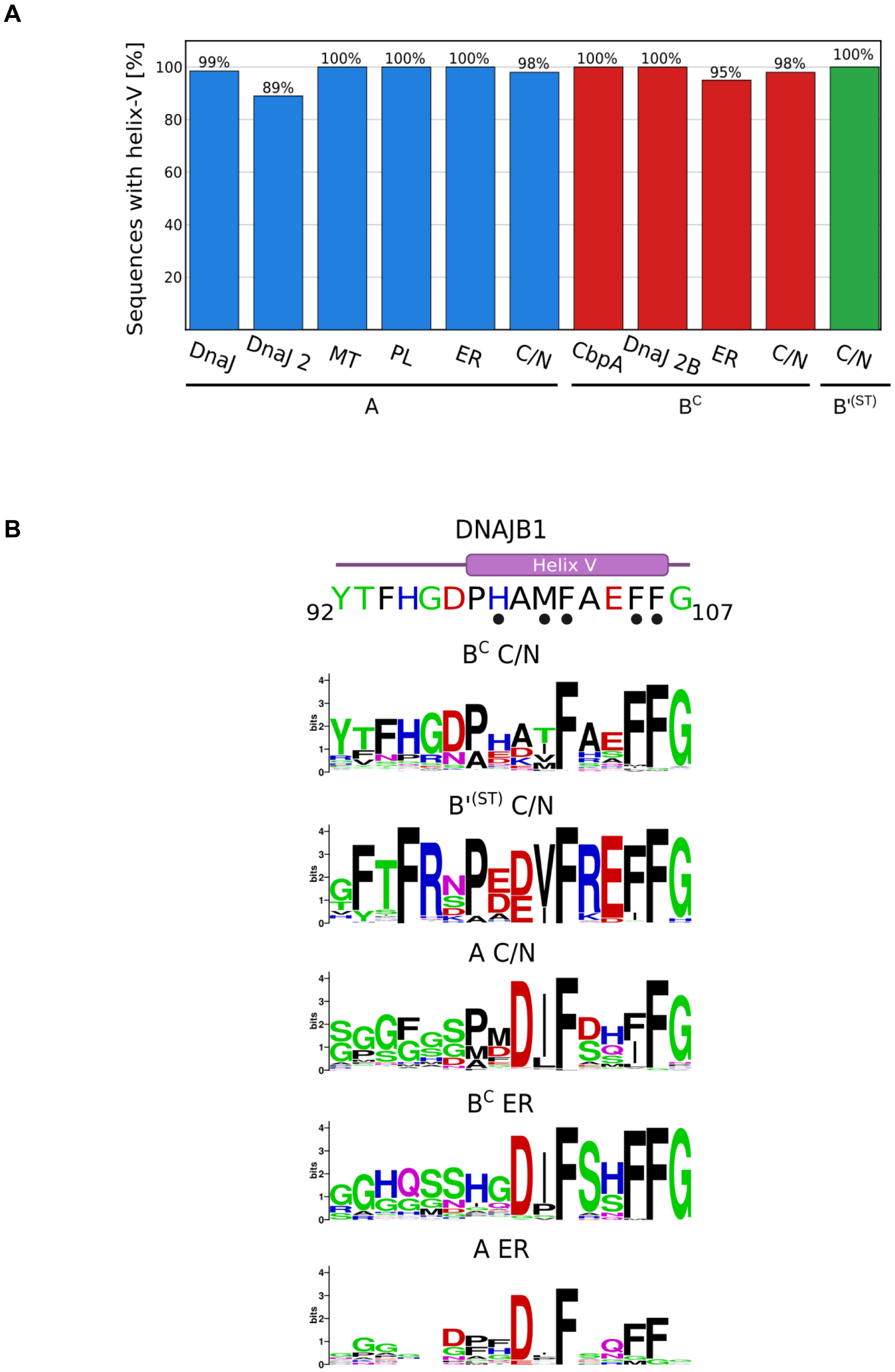


**Figure S27**. Molecular features of the G/F helical segment homologous to helix V of DNAJB1. (A) Presence of helix V across class A, $B^C$, and $B'^{(ST)}$ JDPs. (B) Helix V sequence conservation. (top) Helix V and upstream sequence of DNAJB1; residues critical for autoinhibitory activity are indicated by dots. (bottom) Sequence logos generated from the $AB^CB'^{(ST)}$-alignment.
